# Distinct seasonal infectious agent profiles in life-history variants of juvenile Fraser River Chinook salmon: An application of high-throughput genomic screening

**DOI:** 10.1371/journal.pone.0195472

**Published:** 2018-04-19

**Authors:** Strahan Tucker, Shaorong Li, Karia H. Kaukinen, David A. Patterson, Kristina M. Miller

**Affiliations:** 1 Fisheries and Oceans Canada, Nanaimo, BC, Canada; 2 Fisheries and Oceans Canada, Science Branch, Cooperative Resource Management Institute, School of Resource and Environmental Management, Simon Fraser University, Burnaby, BC, Canada; 3 Forest and Conservation Sciences, University of British Columbia, Vancouver, BC, Canada; INRA, FRANCE

## Abstract

Disease-causing infectious agents are natural components of ecosystems and considered a major selective force driving the evolution of host species. However, knowledge of the presence and abundance of suites of infectious agents in wild populations has been constrained by our ability to easily screen for them. Using salmon as a model, we contrasted seasonal pathogenic infectious agents in life history variants of juvenile Chinook salmon from the Fraser River system (N = 655), British Columbia (BC), through the application of a novel high-throughput quantitative PCR monitoring platform. This included freshwater hatchery origin fish and samples taken at sea between ocean entry in spring and over-winter residence in coastal waters. These variants currently display opposite trends in productivity, with yearling stocks generally in decline and sub-yearling stocks doing comparatively well. We detected the presence of 32 agents, 21 of which were at >1% prevalence. Variants carried a different infectious agent profile in terms of (1) diversity, (2) origin or transmission environment of infectious agents, and (3) prevalence and abundance of individual agents. Differences in profiles tended to reflect differential timing and residence patterns through freshwater, estuarine and marine habitats. Over all seasons, individual salmon carried an average of 3.7 agents. Diversity changed significantly, increasing upon saltwater entrance, increasing through the fall and decreasing slightly in winter. Diversity varied between life history types with yearling individuals carrying 1.3-times more agents on average. Shifts in prevalence and load over time were examined to identify agents with the greatest potential for impact at the stock level; those displaying concurrent decrease in prevalence and load truncation with time. Of those six that had similar patterns in both variants, five reached higher prevalence in yearling fish while only one reached higher prevalence in sub-yearling fish; this pattern was present for an additional five agents in yearling fish only.

## Introduction

Infectious disease is known to influence the survival and evolution of wild animals [1, 2], but has been especially difficult to study in the aquatic realm due to logistical constraints inherent in sampling wild populations of fish over time and the tendency for severely infected individuals to disappear [3]. This is compounded as well by the need for an effective and efficient means of screening for multiple disease agents at once.

Given their high economic value in directed wild fisheries and aquaculture, salmon have been the focus of intensive studies on disease processes worldwide (reviewed in [4]). The study of salmon in the wild is complex given their highly migratory nature and use of different habitats at different stages of life [5] ultimately passing through multiple “infection-scapes” at varying degrees of physiological stress and likely susceptibility [3].

Current knowledge of infectious diseases in salmon is primarily derived from observations of cultured fish, where clinical signs and mortality are observable in freshwater (FW) hatcheries and ocean net pens. However it is not clear that the acute diseases most impactful to fish in high density culture, where transmission is rapid and sub-lethal effects less important, would be among those most significant to the productivity of wild fish populations. We expect that for migratory populations, direct mortality from disease may be less ecologically relevant than indirect effects of chronic disease on physiological performance (swimming, visual acuity, behaviour) that can leave fish vulnerable to predation and other stressors [4, 6]. In fact, mass mortalities due to rapid onset of acute diseases have only rarely been observed in wild migratory fish (e.g. [7, 8, 9]). As a consequence, despite the knowledge that upwards of 90% of juvenile salmon can die in their first summer and fall in the ocean, setting cohort trajectories and stock abundances [10], because mortality is not actually observed, few studies have been carried out to assess the potential role that infectious agents and diseases may play in these substantial losses.

Most infectious agents have never been assessed in migratory juvenile Pacific salmon. Moreover, those studies that have been carried out generally focus on parasites, observable under a microscope [6, 11, 12, 13], or PCR amplification of one to a small number of select agents known to occur within the ecosystem under study [6, 14]. Rarely do studies consider co-infections (but see [6, 15]) or agents associated with emerging diseases in other parts of the world. The application of traditional diagnostic approaches that start with disease investigations of dead or dying fish is difficult and generally intractable in wild migrating fish. Novel molecular genetic tools and techniques employed for high-throughput surveillance of animal populations present a powerful alternative to classical labor-intensive methods of infectious agent screening [16, 17]. These allow for the quantitative characterization of infectious agents and identification of infectious agent carrier states, prior to any potential manifestation of disease symptoms [4, 18]. Specifically, the BioMark^TM^ HT microfluidics Platform can conduct duplicate assays to quantitatively asses the levels of 45 different infectious agents known or suspected to cause disease in salmon (a few are recently discovered but not well studied) [4, 19]. The infectious agents under surveillance include viruses, bacteria, fungi, and protists. This platform, and the agent assays it was populated with, underwent a comprehensive analytical evaluation of efficiency, sensitivity, specificity, and repeatability to ensure that the quantitative infectious agent data used for research purposes are reliable and accurate, and the potential for false positive results are minimized [19, 20].

High-throughput screening by microfluidics qPCR, as applied herein, is an efficient, rapid and reliable means of determining the prevalence and load of multiple infectious agents in a large number of samples at once [4, 20]. We can therefore gain a broad understanding of the prevalence and abundance of *potential* disease causing agents fish encounter upon leaving natal rearing areas. Epidemiological assessments to resolve the temporal and spatial variation of agents can identify those that show the shifting patterns of prevalence and abundance consistent with active replication and/or loss. As a starting point, we define the infectious agents of greatest interest as those displaying concurrent decrease in prevalence and load truncation. Truncation in load distribution is of interest since in macro-parasitology, truncation of over-dispersed loads is often interpreted as evidence of mortality [21, 22]. This is based on the notion that parasite abundance or loads within a population should follow a negative-binomial distribution; a break from the theoretical distribution (i.e. absence of observations at high loads) is defined as truncation. Load truncation and decrease in prevalence may indeed be indicative of mortality but might also reflect an immune response and/or recovery [23]. From there, one can ultimately broaden the scope to assess physiological impacts of agents through direct assessments at the molecular, protein, and cellular levels of individuals with high agent loads (which one cannot assume are diseased, but carry a higher probability of impact) [4], tracking and stress challenge holding studies to assess cumulative impacts with other stressors and linkages with survival (e.g. [17, 24, 25]), and laboratory challenge studies to directly link agents with disease (reviewed in [26]). Ultimately, this approach can open the black box of infection-driven mortality for wild fishes, and salmon in marine environments in particular, and begin to build upon our knowledge of the ecological and evolutionary importance of disease processes, alone or in concert with other stressors.

Chinook salmon are the most diverse and complex species of Pacific salmon with respect to variation in life-history types and consequent residency and migration patterns [5]. These differences are also manifested at the stock level [27, 28, 29] adding another layer of complexity. The Fraser River is the largest river by volume and length (1375 km) flowing into the Pacific coast of British Columbia and is a major natal rearing system for juvenile Pacific salmon including Chinook salmon [5]. Chinook salmon are widely distributed throughout the Fraser River, spawning and rearing from near the river mouth to the headwaters and populations reflect the diversity of their natal system in terms of their genetics and ecology [30, 31]. Populations within the Fraser River system are readily identifiable by genetic stock identification [31]. These many stocks display diverse and contrasting phenotypic life-histories with respect to the timing of juvenile FW residence and adult river re-entry and spawning [30]. Juveniles can enter the ocean directly following emergence in spring, or later during that first summer (ocean-type life-history; aka sub-yearling), or reside in FW for a year or longer (stream-type or yearling). Consequently yearling fish are much larger than sub-yearling fish at ocean entry. Although we lack specific information on Fraser River Chinook salmon as they move into nearshore estuarine habitats, work in other systems denotes the importance of this habitat during this life stage. Juvenile Chinook salmon can rear for periods of weeks to months in estuaries [32, 33]. Distributions of juveniles can be differentiated in the nearshore, shallow brackish habitats by stock and life history type [34]; although it is complex, as a generalization, sub-yearling fish are residing in estuarine habitats longer than yearling fish. The subsequent larger scale migratory trajectory of ocean residence for Fraser river origin fish has been previously defined [27, 28, 29, 35]. Stocks primarily remain resident within the Salish Sea until their first winter, when they begin to move to shelf waters off the west coast of Vancouver Island. They subsequently disperse from there by the second summer of marine life. The exception is some yearling fish which quickly disperse north following ocean entry [27, 28]. Differences in body size between life history types as they transition to the marine environment as well as divergence in dispersion patterns at multiple scales is reflected in other aspects of their ecology. Specifically, Chinook salmon move from a diet of insects and amphipods (nearshore) to crab larvae and fish as they grow and move offshore [36]. Given the larger size of yearling fish and size dependent prey selection, diets are expected to vary between the two life-history types [36, 37, 38], with yearling fish moving to a piscivorous diet first in the marine environment. These diet differences in the early marine period are likely impacting infectious agent exposure through the prey field.

The last ~15 years have seen most Conservation Units (CUs) of Chinook within the Fraser River decline in numbers; the exceptions are sub-yearling CUs whose status has been assessed [39]. Although the potential causes of these declines remains unclear, disease (and carrier states of causative agents) is of interest as a putative factor (see [40]). Different residence patterns of life history variants in both FW and saltwater (SW) as well as their foraging ecology may influence potential exposure to infectious agents ancillary to any hypothesis of stock level effects of disease or carrier states.

Here we contrast the seasonal variation in pathogenic infectious agent prevalence and abundance (i.e. load) in the two life-history types of juvenile Chinook salmon sampled in their first year of life in the marine environment from the Fraser River system. This includes FW hatchery origin fish and samples taken at sea between ocean entry in spring and over-winter residence in coastal waters. Based on known differences in timing of migration, habitat utilization and diet we hypothesized differences in agent profiles between life history types. For the purposes of this study, we refer to the entire suite of infectious agents observed at an overall prevalence of >1% in the total sample as an individual fish’s infectious agent profile. Given the novelty of this multivariate dataset, and subsequent lack of precedence, we borrow some analytical approaches from the community ecology literature to describe the assemblages of infectious agents observed in fish along portions of their migratory trajectory. Furthermore, based on seasonal trends in prevalence and abundance, we attempt to define a transparent means of identifying important or relevant infectious agents as a benchmark moving forward; to tease out which infectious agents carry the epidemiological profiles most consistent with a propensity to contribute to variation in survival during the early ocean period and should be followed up in further studies assessing linkages with molecular, cellular and organismal impacts. It is critical to note that in this initial study, we are not dealing with disease *per se* but rather the abundance and prevalence of potentially pathogenic infectious agents.

## Methods

### Ethics statement

Juvenile salmon were collected under a scientific fishing permit (MECTS # 2014-502-00249) issued to Pacific Region Department of Fisheries and Oceans (DFO) staff by the Government of Canada, DFO, Regional Director Fisheries Management. This work does not require an animal care protocol pursuant to an exemption contained in the Canadian Council on Animal Care (CCAC) guidelines applying to fish lethally sampled under government mandate for assessment purposes (4.1.2.2).

### Sample collection and DNA stock allocation

Analysis centered on juveniles in their first complete year as smolts from FW to ocean residence. Smolt samples were obtained directly from 3 hatcheries (Chilliwack, Middle Shuswap, Spius Creek) within the Fraser River system in spring 2012 collected at the time of release from natal hatcheries, and do not include in river migration. Fish were euthanized in buffered MS-222. These samples are denoted as spring Freshwater (FWSp). Samples of juvenile Chinook salmon in the marine realm were obtained in 4 seasons (spring to winter) between 2008–2012 from various DFO research sampling programs spanning the BC coast − essentially covering a ~ 700 km trajectory of migration during the first year at sea. Fisheries survey methods are summarized in Sweeting et al. [41] and Tucker et al. [28, 35]; all fish were deceased upon net retrieval. Typically, juvenile Chinook salmon were randomly selected from each net tow and fork length (mm) and weight (g) were measured onboard the research vessel. A tissue sample was taken from the operculum and preserved in 95% ethanol for genetic stock identification. We applied the following seasonal size limits to select only juvenile Chinook salmon [28]: May- August < 300mm, Oct-Nov 350mm, Feb-March 400mm.

Fish were allocated to stock of origin according to Beacham et al. [31] using a 268-stock baseline comprised of approximately 50,000 individuals ranging from California to Alaska. Estimated stock of origin of each individual was determined by the probability of assignment to a specific stock or stock. Any individuals with a probability of assignment of < 0.50 were excluded from the analysis. Fish originating from Fraser River stocks were retained for the analysis. Fish were assigned the appropriate life-history type (sub-yearling or yearling) based on stock of origin [30, 40, 42]. Because of uneven sample sizes, we did not conduct statistical analysis on a stock-specific basis. However, given differential FW out-migration timing of South Thompson River fish in particular [29], which dominated sub-yearling samples in some seasons (Table 1), knowledge of stock composition was useful in interpreting results.

**Table 1:** Sample sizes, lengths (mm ± SD) and weights (g ± SD) of sub-yearling and yearling type juvenile Chinook salmon from Fraser River system stocks sampled in different seasons.

	**season**							total	length	weight
		L Fras	S Thom							
Sub-yearling	spring FW	43	30					73	78.6 (4.7)	5.5 (1.1)
	spring SW	7	-					7	123.4 (31.2)	40.8 (25.5)
	summer	3	10					13	121.9 (34.0)	26.2 (24.1)
	fall	5	132					137	141.5 (20.7)	34.3 (22.3)
	winter	5	6					11	243.9 (33.2)	180.4 (77.8)
								**241**		
		L Fras	M Fras	U Fras	N Thom	S Thom	L Thom			
Yearling	spring FW	-	21	-	-	-	-	21	108.6 (7.0)	14.1 (3.0)
	spring SW	3	18	18	7	2	8	56	131.9 (23.9)	39.4 (22.3)
	summer	2	129	77	40	2	40	290	153.4 (24.2)	49.3 (22.4)
	fall	-	15	9	8	6	3	41	204.6 (43.7)	119.2 (66.4)
	winter	-	-	4	-	-	2	6	319.2 (18.3)	398.8 (60.8)
								**414**		

FW = freshwater; SW = saltwater; Fras = Fraser River; Thom = Thompson River; L = Lower; M = Middle; U = Upper; N = North; S = South.

### Infectious agent detection

Multiple tissues (gill, whole brain, liver, head kidney, and heart tissues) were sampled for molecular genetic analysis of infectious agents. These tissues were selected to include the range of primary and secondary infective tissue for the infectious agents on our panel. Tissues were dissected and collected in the field and preserved in RNAlater (Qiagen, MD, USA) for 24 hours at 4°C and then frozen in -80°C. Alternatively, whole fish were frozen immediately at -80°C and dissected in the lab at a later date.

We conducted quantitative RT-PCR of infectious agents using TaqMan assays run on a Fluidigm BioMark^TM^ HT microfluidics platform (S1 Table). The BioMark platform has been evaluated for specificity, sensitivity and repeatability and subsequently analytically validated for research application in salmon [19]. Assays, primers and technical details for running samples on the BioMark platform are outlined in Miller et al. [19]. Briefly, the platform can run 96 assays against 96 samples at once, with each reaction conducted in an individual chamber (9,216 reactions on a single dynamic array). TaqMan assays were run in duplicate, with each dynamic array containing 46 assays to 45 infectious agents (two assays to infectious salmon anemia virus), one housekeeping gene control, and one negative assay control. Each run included 80 samples and 16 controls, including 5 serial dilutions of an artificial construct standard (for quantitation of all infectious agents) and positive and negative processing controls. For analyses herein, trizol homogenates of the five sampled tissues were combined and extracted for RNA and DNA to maximize the detection across a broad spectrum of infectious agents. Technical details for nucleic acid preparation are in Miller et al. [43] and for the BioMark are presented in Miller et al. [4, 19].

Cycle threshold (CT) was determined using the BioMark Real-Time PCR analysis software. Reaction curves for each positive sample–assay combination were visually evaluated for abnormal curve shapes, close correspondence between replicates and presence of vector contamination. The real-time qPCR results were exported as table view csv file and the average of the duplicated samples calculated. Samples amplifying products from only one duplicate were treated as negative; negatives were all given a threshold cycle (CT) of 45. In this study, the limit of detection (LOD) is defined as the estimated cycle threshold (CT) number under which we expect true positive results 95% of the time for a given assay [19]. We used a conservative cut-off of CT<27 to score individuals as ‘positive’ or ‘detected’; this equates to a CT of 35–36 on the ABI 7900 and is near the upper limit of reliable repeatability for most assays [19]. The evaluation of reproducibility demonstrated the 10 CT difference between platforms [19]. To make the data more amenable to those not familiar with qPCR (where a lower cycle threshold (CT) indicates a higher quantity of genetic material), we chose to express measures of infectious agent RNA quantity as ‘relative load’ instead of CT. CT’s were transformed to relative CT by subtracting from 45. This inverts the scale such that negatives or absence of an infectious agent is zero. Hereafter we refer to the load or abundance of an infectious agent within a fish as its relative CT score. Infectious agent prevalence was calculated across all samples (percentage of fish with a positive infectious agent detection). Given sample size concerns, we only considered infectious agents which had a prevalence of >1% in subsequent analysis. Subsequently, infectious agent prevalence was calculated for life history and season groups. Diversity was calculated as the sum of all positive detections across the infectious agents at >1% prevalence (i.e. number of infectious agents per fish).

### Statistical analysis

All analyses were conducted using R-based packages (R® version 3.2.5 [44]).

#### Infectious agent prevalence, load and diversity

Permutation ANOVAs were applied to test for differences in mean prevalence, load and diversity between infectious agents sampled in the different seasons and life history types using the LmPerm package in R [45].

#### Trends in individual infectious agent prevalence and load

We statistically defined the seasonal shifts in prevalence through simple linear models of presence-absence across samples using least square means (LS means [46]). This allows one to compare group responses when the data are unbalanced. When data are unbalanced, using simple averages are not appropriate since all factors do not have an equal chance to affect the response. LS means are generalizations of covariate-adjusted means based on predictions from a model over a grid of predictor values. More explicitly, a set of reference levels is defined for each predictor, and a grid is created consisting of all combinations of these. Predictions are made on this grid, and, LS means are computed marginal means of those predictions, using equal weights. Custom, orthogonal contrasts between life history types and consecutive seasons were constructed to evaluate the statistical significance of trends over time. Confidence intervals (CIs) and comparison arrows were plotted to visualize statistical trends using the Lsmeans package in R [46]. Side-by-side confidence intervals are useful to understand the range of values but are not appropriate for comparison between groups in this case. This is because the standard errors of means are not the same as the standard errors of *differences* of means. Therefore to visualize differences between groups we also plotted “comparison arrows” as generated in the lsmeans package. These are derived from the lengths of the confidence intervals for the differences between pairs of means, and consequently how much these intervals overlap zero (difference in means). Accordingly, a measure of "covering zero" is computed (the more an interval covers zero, the greater it is, and the more it doesn't, the more negative it is). The algorithm uses weighted least-squares to fit the lengths of the arrows (as regression parameters) so that the overlap values match the zero coverages. The method is *ad hoc*, but works effectively as a means of visualization of statistical trends [46]. P values were adjusted for multiple comparisons using the Benjamini-Hochberg procedure [47].

**Fig 1:**
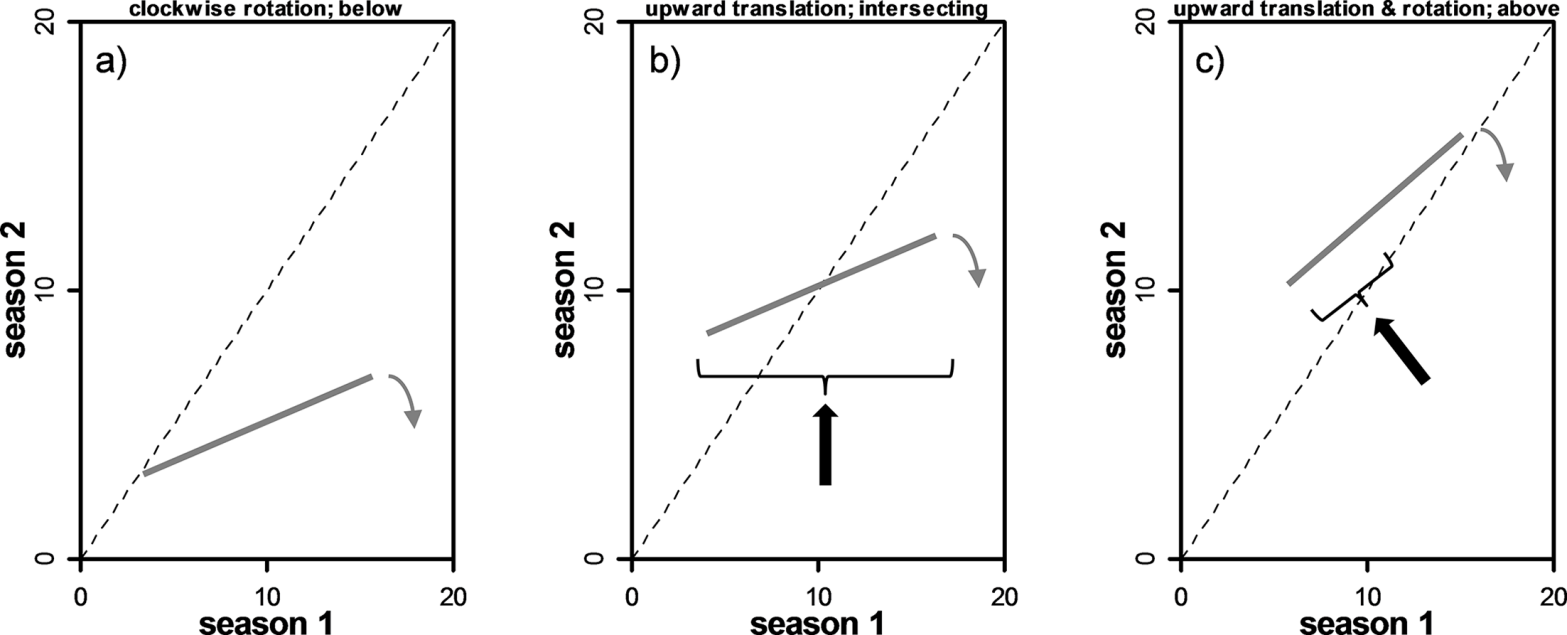
Examples of three scenarios for detecting loss of relative high loads of infectious agents using quantile-quantile plots. Load distributions are contrasted between sequential seasons. For all three scenarios, the slope is significantly less than 1 (i.e. 1:1 line; dashed line). The different scenarios account for any potential infectious agent dynamics in the host and the consequent displacement relative to the 1:1 line (where loads would be similar between seasons): a) no seasonal incremental change in loads overall; b) an equal seasonal incremental increase in all loads; c) an incremental increase in small loads that is higher relative to larger loads. Grey, arcing arrows show the rotational effect of selective high load loss; black arrows and brackets denote the translational effect of increases in load between season 1 and season 2.

Subsequently trends in infectious agent loads (relative CT) were explored to evaluate whether relative high loads became truncated at any point. This was not based on *a priori* notions of what constitutes a high or low load (i.e. some absolute load level). Rather, changes in the frequency distribution of loads were contrasted over time. Nor were assumptions made about the form and fit of the stock distribution (e.g. negative binomial vs. Poisson) [21]. An empirical quantile-quantile plot can be used for comparing two probability distributions by plotting paired-percentiles from these distributions [48]. Empirical quantile-quantile plots were used to determine whether or not relative high level loads persisted or the distribution became truncated in consecutive seasons. Loss or truncation of high loads would be denoted by quantile-quantile slopes significantly smaller than one, and one of the following theoretical scenarios (based on the possibility of infectious agent dynamics within the stock): a clockwise rotation below the 1:1 line (when there is no seasonal incremental change in loads overall); upward translation while intersecting the 1:1 line and a clockwise rotation (when there is an equal seasonal incremental increase in all loads). If there is an incremental increase in small loads that is higher relative to larger loads we would see a tendency for the distribution to be above the 1:1 line (upward translation) but with countervailing rotations in the case of truncation. Scenarios of interest are conceptually illustrated in Fig 1. We regressed the 10, 25, 50, 75, 90th load percentiles within a season as a function of the same percentiles for the previous season. This analysis was done separately for all pairs of sequential seasons. Because load-frequency distributions may not be adequately described with low sample size, we used a bootstrap procedure to estimate the load at these percentiles and the confidence intervals associated with the quantile-quantile slope. Briefly, seasonal loads were independently resampled with replacement from their respective distribution to generate a pseudo load-frequency distribution for each season. We then used these pseudo load-frequency distributions to estimate the slope of the quantile-quantile plot using the 10, 25, 50, 75, 90th percentile of sequential seasonal loads. This procedure was repeated 10,000 times. The mean and 95% confidence limits of the quantile-quantile slopes were taken as the 50th percentile, and the 2.5th and 97.5th percentile, respectively. This analysis was performed using the bootstrap package [49]. There are caveats to analysis; sample size is not even and the number of fish is generally lower in spring, fall and winter. Moreover, we are assuming that hatchery origin fish from FW spring are representative of the state of wild stocks.

#### Multivariate analysis: Infectious agent profiles among individual fish

Co-occurrence of infectious agents was evaluated after transforming relative CT to binary presence-absence. To visualize the associations, infectious agent profiles were partitioned by clustering using a fuzzy c-means clustering approach (function fanny; R package cluster [50]). Recognizing that cluster limits may not be absolute, this method associates all objects a series of values measuring the strength of the membership to various clusters. An object that is clearly linked to a given cluster has a strong value and weak or null values for other clusters (sum up to 1 for each object). The clusters are visualized by a silhouette plot.

Permutation MANOVA (PMANOVA) was applied to test the multivariate infectious agent profiles (relative CT scores) for season and life history effects (adonis function, Vegan Community Ecology Package Version 1.17–8 [51]). PMANOVA is a non-parametric version of MANOVA except that it uses distance matrices to partition sums-of-squares and permutations to develop pseudo-F ratios to determine the significances of those partitions. Distance matrices were constructed using Bray Curtis dissimilarities; we used 10,000 permutations. In so far as it partitions the sums of squares of a multivariate data set, adonis is directly analogous to MANOVA and is a robust alternative to both parametric MANOVA and to ordination methods for describing how variation is attributed to different predictors or covariates.

#### Spatial effects on infectious agent profiles

Sample locations of juvenile Chinook salmon were mapped out in each season. Distribution maps were generated using the PBS Mapping package in R [52]. We used the geoXY function (R package SoDA [53]) to return the corresponding relative coordinates in X (east-west) and Y (north-south) distances (km) along the surface of the earth relative to a point at the mouth of the Fraser river as a specified origin. To assess spatial correlations in infectious agent profile composition between sampling sites, we constructed a Mantel correlograms on a seasonal basis [54, 55] using the “mantel.correlog” function within the Vegan version 2.0–5 Library in R [51]. In brief, the Mantel's test of matrix correlation, *r*_*M*,_ is calculated at multiple distance classes and plotted across the full range of distances. In those distance classes where *r*_*M*_ is positive and significantly different than zero, the multivariate similarity among samples is higher than expected by chance, or less similar, showing a negative autocorrelation [54]. The significance of the correlogram was tested with 10,000 permutations.

## Results

### Overview: Infectious agent prevalence, load and abundance

**Fig 2:**
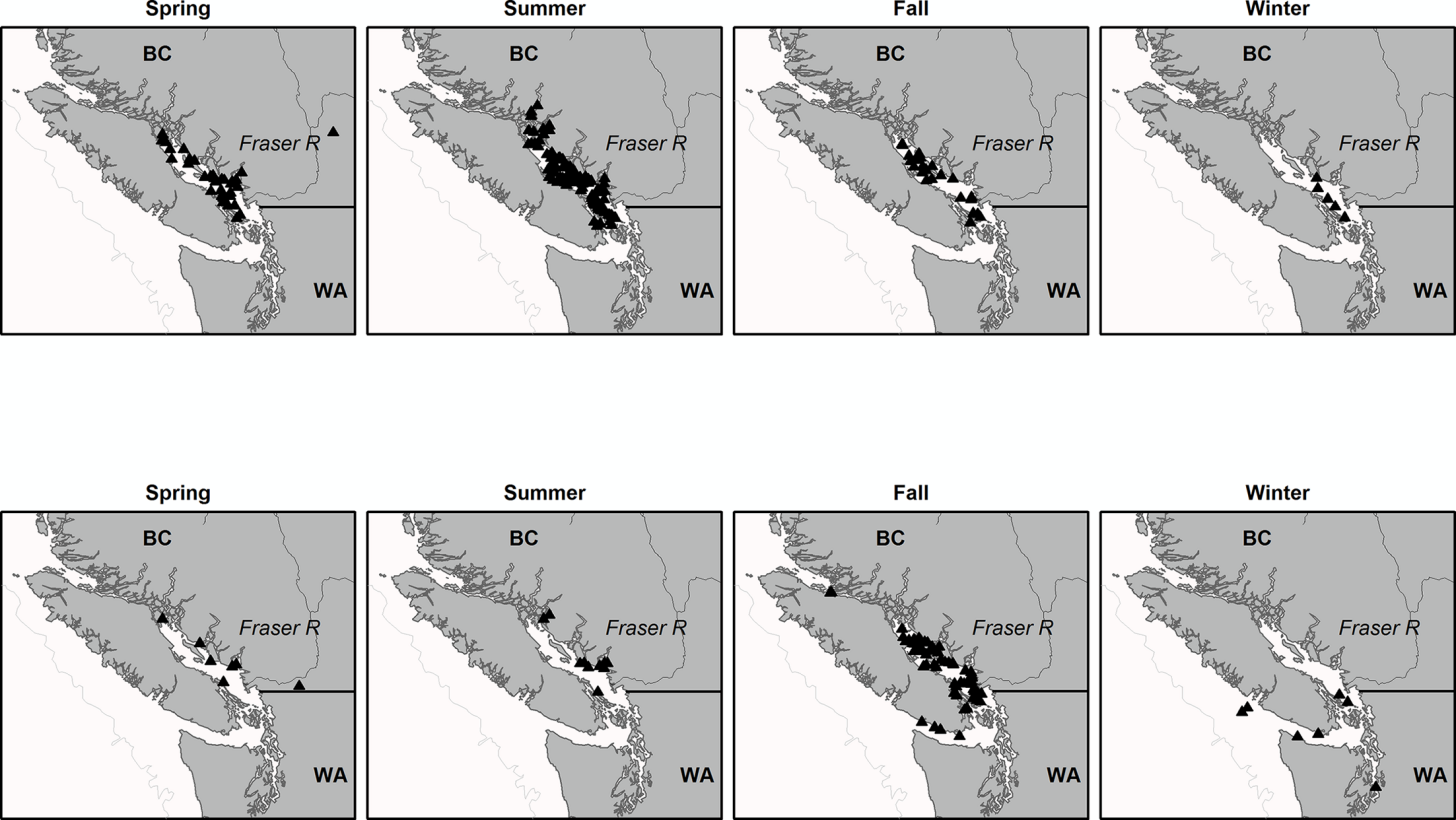
**Catch locations of yearling (a) and sub-yearling (b) Fraser river origin juvenile Chinook salmon.** Salmon were caught between 2008–2012.

**Fig 3:**
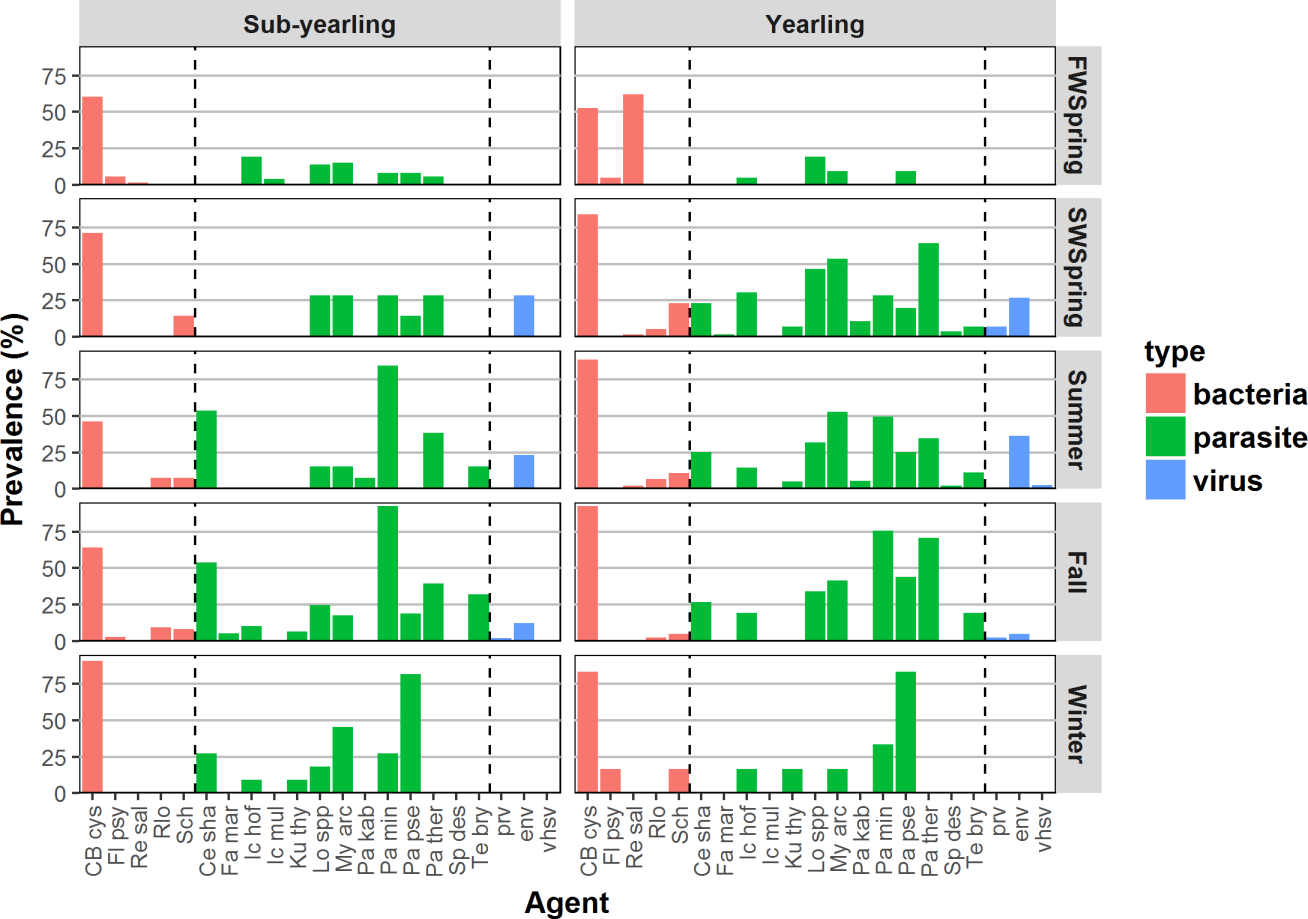
Seasonal prevalence of infectious agents in sub-yearling and yearling juvenile Chinook salmon from the Fraser River. Sample sizes are provided in Table 1; full names are provided in S1 Table. Infectious agents are separated into type (bacteria, parasite and virus).

655 juvenile Chinook salmon allocated to Fraser River system stocks (Table 1) were caught primarily in the Strait of Georgia (Fig 2). Of the 45 infectious agents in the screening panel, 32 had positive detections. However, only 21 infectious agents had an overall prevalence >1% (S2 Table). These included 3 viruses, 5 bacteria and 13 parasites. Nine infectious agents had an overall prevalence >10%. Six infectious agents are thought to be transmitted in fresh water, six in salt water, and nine in both environments. Overall prevalence (Fig 3) ranged from 1.0% (ic.mul) to 78% (c.b.cys) and varied by infectious agent (p <0.0001), season (p <0.0001) and life history (p = 0.006). Sub-yearling fish demonstrated a trend of slightly higher prevalence of FW-transmitted infectious agents. Under least-square means analysis, the prevalence of 7 individual infectious agents varied significantly by life-history type and season (S3 Table). These included c.b.cys, ce.sha, my.arc, pa.min, pa.pse, pa.ther and re.sal (see S2 Table for full agent names). Eight agents varied significantly by season only (S3 Table); fa.mar, fl.psy, lo.sal, pa.kab, prv, sch, te.bry and env.

**Fig 4:**
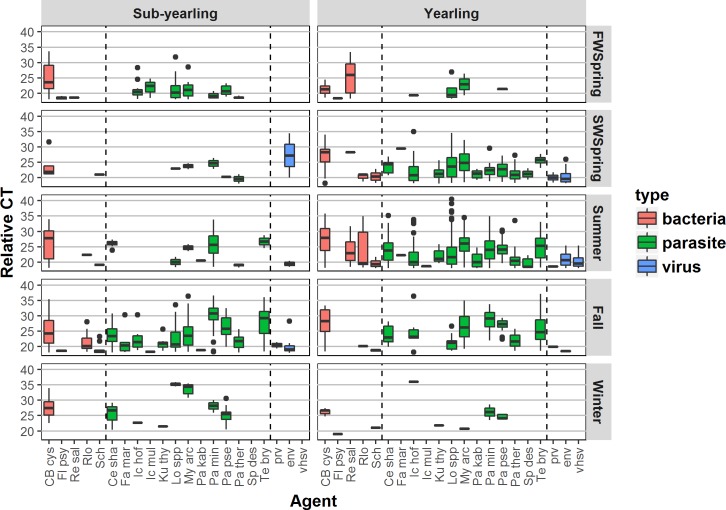
Boxplot of seasonal load (relative CT) of infectious agents in sub-yearling and yearling juvenile Chinook salmon from the Fraser River. Sample sizes are provided in Table 1; full names are provided in S1 Table. Infectious agents are separated into type (bacteria, parasite and virus).

Positive agent loads (relative CT; Fig 4) ranged up to 36 (where CT = 9; >100,000 copies per μl) and also varied by infectious agent (p<0.0001) and season (p = 0.011) but not by life history type (p = 0.63). One infectious agent, vhsv, was only detected in a single season in 8 yearling fish.

**Fig 5:**
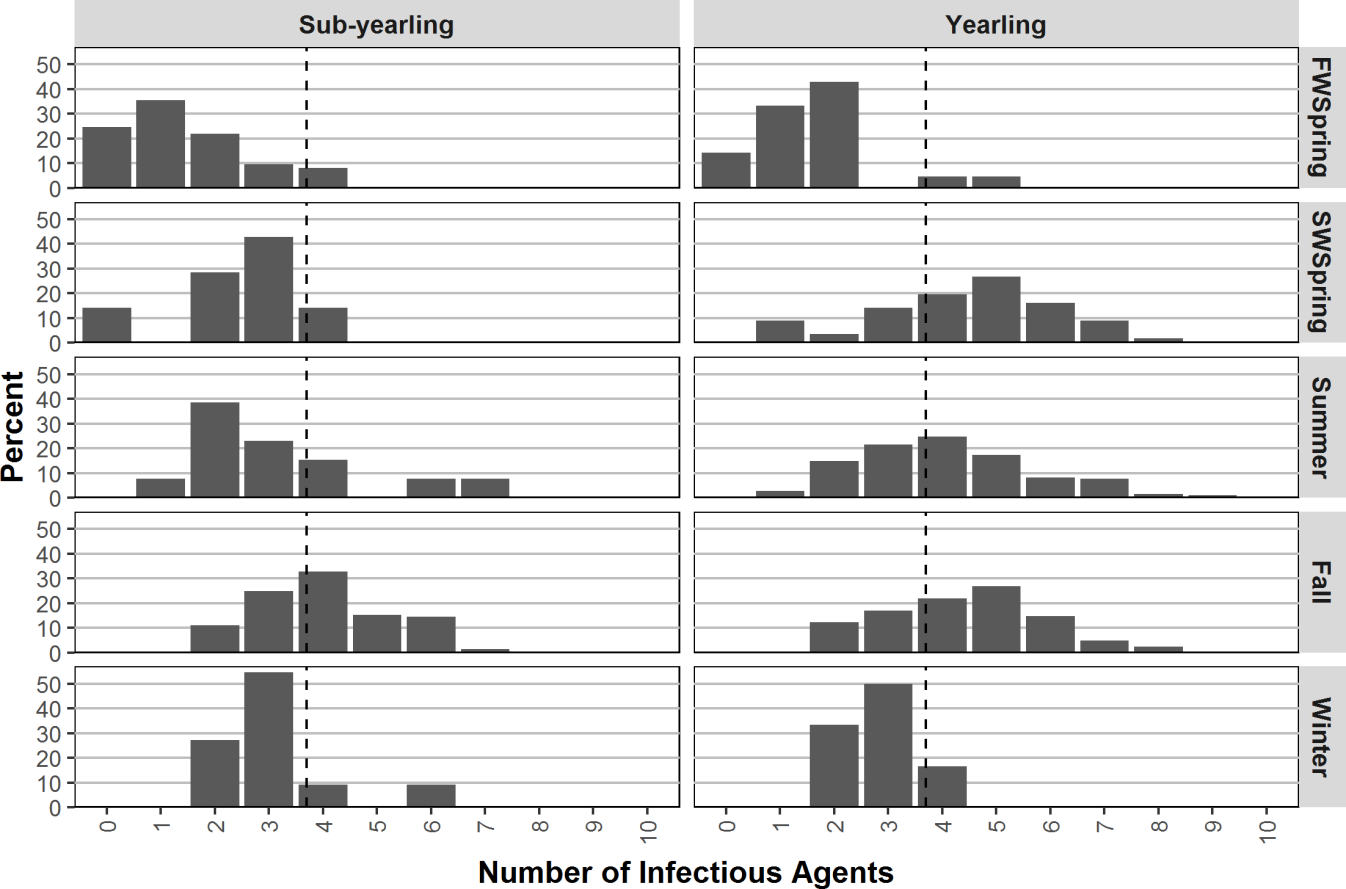
Seasonal infectious agent diversity (% individuals) with 0–10 agent detections for sub-yearling and yearling Chinook salmon. Dashed reference line represents pooled sample mean of 3.7 infectious agents.

Infectious agent diversity ranged from 0–10 per individual fish (Fig 5). Twenty-one fish sampled in FW hatcheries were agent “free”, and two fish sampled in summer SW had no infectious agents; in all other seasons, fish had at least one infectious agent. Over all seasons, juvenile Chinook salmon carried on average of 3.7 infectious agents. Diversity changed significantly between seasons (p<0.0001), increasing upon SW entrance, increasing through the fall and decreasing slightly in winter (Fig 5). Diversity varied between life history types (p = 0.008) with yearling individuals carrying 1.3-times more infectious agents on average; the interaction between life history and season was significant (p = 0.04) suggesting diversity changes differentially between the two types (Fig 5). Statistical results (not shown) were similar when considering SW origin infectious agents exclusively; yearling individuals had 1.5-times higher diversity of SW origin infectious agents. Stream- and sub-yearling fish had similar diversity of FW origin infectious agents.

### Individual infectious agents: Trends in prevalence and load

Below we summarize the trends for individual infectious agents displaying significant seasonal differences in prevalence. Statistical results are presented in the supplementary material (S3 Table). Load truncation is noted where significant (S4 Table). Agents are grouped based on whether 1) both life-history variants displayed concurrent declines in prevalence and load truncation, 2) this same pattern was observed in only one of the variants or 3) displayed some other significant seasonal pattern in prevalence.

#### Decline in prevalence coupled with load truncation in both life history types

*Parvicapsula minibicornis* (pa.min): FW transmitted, but large increases in prevalence were observed for both types in SW spring (from 0–8% to 29%). Thereafter both sub-yearling and yearling saw increasing prevalence till fall (sub-yearling 93%; yearling 76%) and declined abruptly in winter (sub-yearling: 27%; yearling: 33%). Load truncation was observed in both types between fall and winter.
*Paranucleospora theridion* (pa.ther): SW (sea lice transmitted) origin (in Norway) but two weak detections (CT>26; <10 copies per μl) were observed in FW in sub-yearling. In yearling fish, there was a large increase in prevalence upon SW entry to 64%, declined to 39% in summer, then increased to 71% in fall and declined abruptly again to 0% by winter. Sub-yearling fish also saw increases in prevalence in SW peaking at 39% in fall and also declining abruptly to 0% by winter. Loads were therefore truncated in both types in winter.
*Piscine orthoreovirus (*prv): Transmitted in both environments. In yearling fish, prevalence increased from 0% in FW to 7% in SW spring and declined thereafter between 0% (summer, winter) and 3% (fall). A prevalence of 2% was observed in sub-yearling fish in fall. Loads became truncated with 0% prevalence in winter.
Salmon gill *Chlamydia* (sch): Only previously studied in SW (in Norway), was absent in FW. In sub-yearling fish, prevalences remained between 8–14% before declining to ~0% in winter. In yearling fish, prevalence was 23% in SW spring, declining to 11% and 5% respectively in summer and fall, and increasing again to 17% in winter. Load truncation was observed in yearling between summer and fall (during the decrease in prevalence) and in both types between fall and winter.
*Tetracapsuloides bryosalmonae* (te.bry): Transmitted in both environments. Prevalence increased from 0% in FW peaking in fall for both types (yearling ~35%; sub-yearling 20%) thereafter abruptly dropping to 0% in winter. Loads were therefore truncated in both types in winter.
*Erythrocytic necrosis virus* (env): SW origin. Prevalence increased significantly to ~27% in SW spring in both types. Sub-yearling prevalences remained at this level while yearling increased to 37% then declined for both in fall and disappeared by winter. Load truncation was observed in yearling between summer and fall and in both types between fall and winter.

#### Decline in prevalence coupled with load truncation in one life history type

*Candidatus Branchiomonas cysticola* (c.b.cys): Only previously studied in SW, but was present in FW at high prevalence (sub-yearling 60%, yearling 52%) and increased in SW spring to 71% in sub-yearling and 84% in yearling. Thereafter, prevalence increased to 93% in yearling fish in fall and declined to 83% in winter. Prevalence declined in sub-yearling fish in summer to 46% and increasing again to 91% in winter. Load truncation was observed in both sub-yearling and yearling fish between fall and winter.
*Ceratonova shasta* (ce.sha): FW origin endemic, but was absent from FW fish sampled at hatcheries. The parasite was observed at high prevalence in SW, peaking at 54% in sub-yearling and 27% in yearling in the summer or fall, with prevalence declining to 27% by winter respectively. Load truncation was observed in yearling fish only between summer and fall and in winter as prevalence declined to 0%.
*Loma spp* (lo.spp): Prevalence increased by >2 fold with SW entry in both types although types diverged in summer and fall (yearling > sub-yearling). Prevalence in yearling declined thereafter dropping to 0% by winter; prevalence’s in sub-yearling fluctuated between 15–25%. Load truncation was observed in yearling fish between summer and fall and in both between fall and winter.
*Myxobolus arcticus* (my.arc): FW transmitted but prevalence increased in SW spring in both types although types diverged (yearling > sub-yearling) then remained generally stable till winter where there was a decline in yearling to 15% and an increase in sub-yearling to almost 50%. Load truncation was observed in both types between fall and winter.
*Renibacterium salmoninarum* (re.sal): High prevalence found in FW spring in yearling only. Prevalence dropped off abruptly in SW to 0% by fall. Loads were therefore truncated by fall.

#### Other significant seasonal pattern in prevalence

*Flavobacteria psychrophilum* (fl.psy): FW origin. Initial decline in prevalence in SW in both types (from ~5 to 0%) but resurgence occurred in winter in yearling fish with prevalence’s of ~18%.
*Parvicapsula pseudobranchicola* (pa.pse): SW transmitted with prevalence’s peaking in winter (~80%) in both types. However load truncation was observed in yearling fish in winter.

### Infectious agent profiles (multivariate analysis)

#### Infectious agent associations

**Fig 6:**
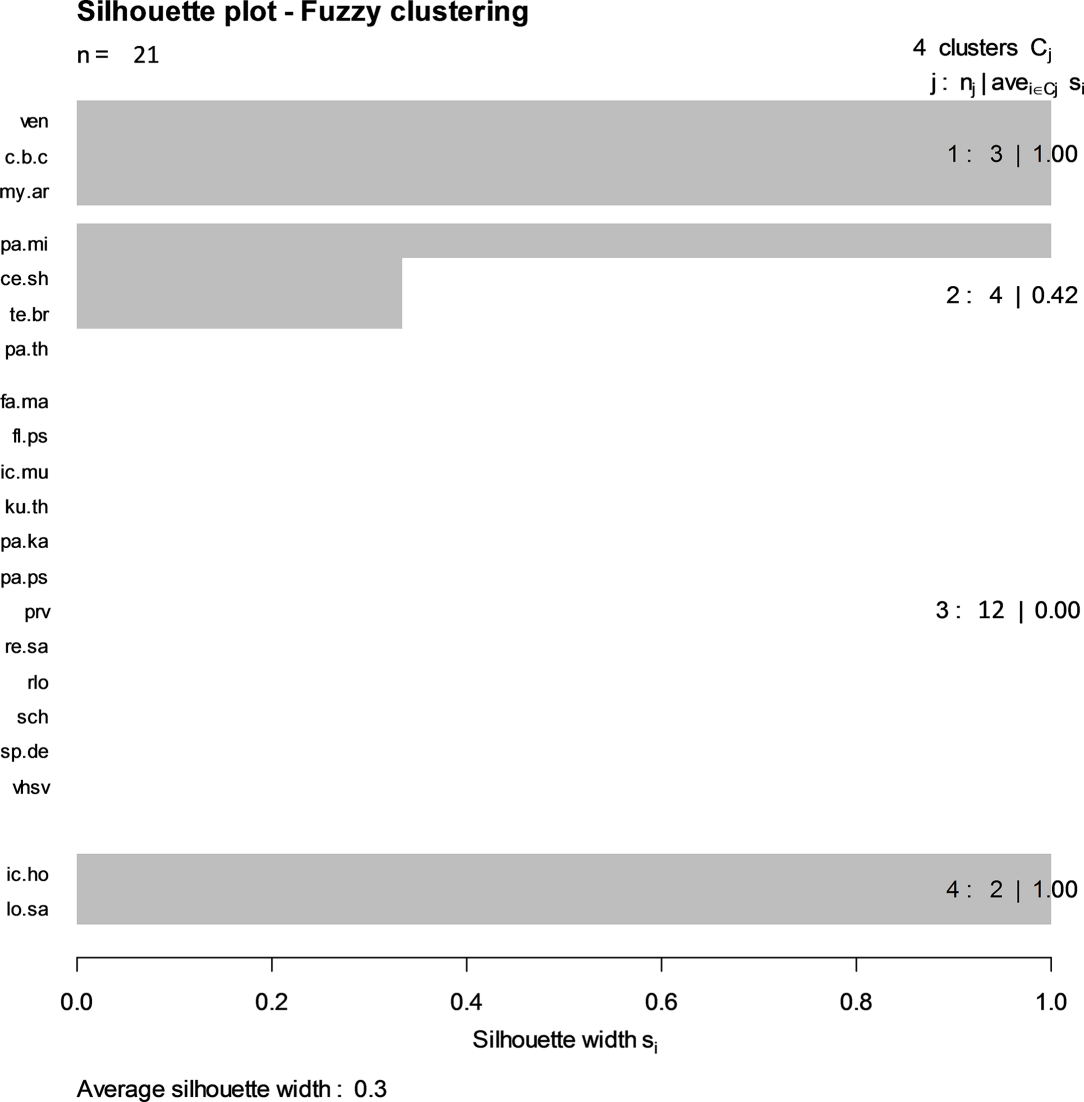
Silhouette plot of fuzzy c-means clustering of infectious agent profiles in juvenile Chinook salmon. The number of infectious agents in each cluster and the corresponding mean membership score (silhouette width) is noted. The average silhouette width was 0.3 denoting relative weak association overall.

There were four main clusters, three showing positive associations between infectious agents: 1) env, c.b.cys and my.arc, 2) pa.min, ce.sha and te.bry, and 3) lo.sal and ic.hof, (Fig 6). However, the associations were generally weak overall (average cluster membership score = 0.3).

#### Permutation MANOVA

While season and life history were significant, these only explained 20% of the variation in infectious agent profiles (Table 2). Season explained the highest amount of variation (17%).

**Table 2:** Permutation (10,000) MANOVA results for season and life history effects on infectious agent (n = 21) profiles.

	Df	SumsOfSqs	MeanSqs	F.Model	R^2^	Pr(>F)
season	4	25.692	6.4231	33.377	0.165	< 0.001
life history	1	2.42	2.4198	12.574	0.016	< 0.001
season: life history	4	3.798	0.9495	4.934	0.024	< 0.001
Residuals	645	124.122	0.1924		0.796	

#### Mantel’s correlograms

Fish were caught between 6 and 642 km from the mouth of the Fraser River (straight line distance). For any season, there were no significant distance classes suggesting (multivariate) infectious agent profiles show no spatial patterns over the scale of this survey.

## Discussion

Through application of the BioMark platform we detected the presence of 32 infectious agents, 21 of which were at >1% prevalence, in juvenile Chinook salmon in a dataset that spanned the first year of ocean residence. Our study, which contrasts two life-history variants with divergent stock status, represents the most comprehensive survey conducted to date in salmon specifically, or fish generally, in terms of the breadth of infectious agents and temporal scope. While we did not directly establish links between infectious agents and disease, shifts in prevalence and load over the transition to marine residence into the first winter at sea were examined. As predicted, we found that sub-yearling and yearling Chinook salmon carried different agent profile in terms of diversity, the origin or transmission environment of agents, and the prevalence and abundance of individual agents. Subsequently we identified eleven infectious agents displaying concurrent drops in prevalence and load truncation, a pattern that could readily (but not exclusively) be explained by mortality. The dissimilarities in agent profiles may reflect different residency patterns and periods in near-shore and offshore environments and consequent direct exposure to infectious agents or through proximity/contact with intermediate or alternate hosts or alternatively through diet.

In general, sub-yearling fish have limited FW residence, going to sea in their first year of life while yearling fish rear in natal riverine habitats for 1–2 years prior to ocean migration [5]. However, sub-yearling Chinook salmon stocks from the South Thompson system within the Fraser River, which comprised the majority of our sub-yearling sample, display late ocean entry timing relative to other stocks [29], entering the Strait six to eight weeks later than the yearling stocks they were compared to. It is important to note that this entry timing is inferred from the capture or absence of stocks in pelagic surveys (>30 m bottom depth) in the Salish Sea as opposed to explicit sampling in river or the estuary. In the intervening six to eight weeks, it is presumed that sub-yearling fish generally reside in the lower river estuary, potentially enhancing their exposing to certain myxozoan parasites with alternate hosts residing predominately in estuarine environments. Hence, in our study, over the course of the first year of ocean sampling May through March, most yearling fish would have experienced a longer time period in SW. Given their greater size, it is also likely they had a larger diet breadth in that first year at sea [36, 37, 38]. Hence, while, it is possible that differences in infectious profiles represent inherent differences in susceptibility, the differential patterns of infection, with estuarine myxozoans being more prevalent in sub-yearling than yearling stocks and a generally higher diversity of marine-transmitted agents in yearling fish, supports a mechanism of environmental or prey field exposure over susceptibility.

### Infectious agent diversity

Higher diversity and prevalence of SW agents for yearling fish is conceivable given their longer residence in SW during the sample period of our study. We found that average agent diversity was slightly higher for yearling fish (3 Vs 4), due predominantly to higher average diversity of SW-origin infectious agents (1.3 Vs 2). Sub-yearling fish had higher prevalence in 4 of 6 FW-origin infectious agents, including the two myxozoan parasites which share a common host (*Ceratonova shasta* and *Parvicapsula minibicornis*) [56, 57], a protozoan parasite (*Ichthyophthirius multifiliis*) and the bacteria *Flavobacteria psychrophilum*. Yearling fish had higher prevalences of the other myxozoan parasites *M*. *arcticus*, which originates in many FW natal rearing areas [58, 59], and *Parvicapsula kabatai*, largely transmitted in the marine environment [60, 61]. We hypothesize that these differences in prevalence in FW and estuarine agents are largely the result of higher exposure due to longer residence times in the environments in which the specific agents originate. However there might be some finer scale habitat use resulting in life history differences in exposure to alternate hosts. Alternately, yearling fish had higher prevalence in 5 of 6 SW origin and 5 of 6 FWSW origin infectious agents, demonstrating further differences between the variants. The exceptions were *Facilispora margolisi* and *Tetracapsuloides bryosalmonae* respectively. *F*. *margolisi* is a microsporidian recently identified from, and likely transmitted by sea lice [62] with similar prevalence in both life-history types. *T*. *bryosalmonae* (discussed below) is the causative myxozoan agent of proliferative kidney disease (PKD) transmitted in FW from a bryozoan host (*Fredericella sultana*) [63, 64]. Six infectious agents, four parasites (*Paranucleospora theridion*, *Parvicapsula pseudobranchicola*, *Ichthyophonus hoferi*, and *Loma spp*), one bacteria (*Candidatus Branchiomonas cysticola*) and one virus (Erythrocytic necrosis virus;ENV) stood out with the strongest differential in prevalence, with higher levels in yearling fish. This observation may be explained by a higher exposure early on of yearling fish to marine fish species by virtue of higher affinity for more offshore environments [28, 36], and an earlier shift to a piscivorous diet [37]. *P*. *theridion*, transmitted via sea lice, would be expected to be in higher abundance among co-migrating herring [65]. ENV, *Loma spp*, *C*. *B*. *cysticola*, and *I*. *hoferi* are known to be carried by many marine fish species [66, 67, 68]. *I*. *hoferi* is known to be transmitted orally through ingestion of infected prey [69].

On average, 3.7 agent detections were found in individual fish with a maximum of 8–10 in a few individuals. Diversity increased upon SW entrance from 1.5 to 4.2 and was maintained at 4.1 over the seasons till dropping to 3.0 in winter. Interestingly, this average generally exceeded infectious agent diversity of Sockeye salmon smolts predated by a seabird [4]. Therefore, the potential for cumulative and indirect effects of multiple agent infections is plausible as well for juvenile Chinook salmon, although as yet untested. Similar patterns in diversity are observed in other stocks of non-Fraser Chinook salmon utilizing the Salish Sea (Thakur unpublished; Tucker unpublished). At this point we don't know what the fate of any of these individual fish would be. However, these results suggest that agents may play a substantive role in mortality; a component of which may be increased susceptibility to predation (see Case IV in [4]) where predation may reduce the prevalence of an infection agent in the population as a whole [70]. A recent study [71] raises the prospect of increased predation on stocks of threatened juvenile Salish Sea Chinook salmon through increasing populations of pinnipeds. This is to the inferred detriment of a specific endangered killer whale stock (Southern Resident) since adult Chinook salmon are their primary diet item [72]. While increased predation may indeed be true, what is not clear, nor considered, is whether in fact predation by pinnipeds is compensatory or additive. It could be the case that the culling of fish with high infection loads may help curb the spread of infectious agents and consequent mortality in the stock overall. In this case pinnipeds could in fact be providing a net benefit to salmon stocks and dependent predators exploiting salmon in later life-stages. How we manage endangered resources may be profoundly encumbered by an oversimplification of ecological dynamics. We expect that the analytical power of the microfluidics multi-agent qPCR approach has the potential to help answer and interpret complex ecological questions and the role of infectious agents in stock dynamics.

### Seasonal trends in infectious agent prevalence and load

Traditional studies rarely contrast shifts in infectious agent prevalence and load between sampling dates, therefore there is very little precedent for this type of analysis. Over-dispersion (mean to variance ratios) of parasite load is often used as indirect evidence of mortality [11, 73, 74]. Alternately, use of a negative binomial distribution truncation technique described by Crofton [21] has been a widely accepted model for macroparasites (see [75]). These approaches are often used on samples from one time interval. Here, given that we had the benefit of sequential samples, we directly contrasted the frequency distribution of infectious agent loads between seasons. We defined infectious agents of greatest interest as those demonstrating a seasonal pattern consistent with stock-level impacts, notably a drop in prevalence and load truncation. One caveat of this approach is that with macroparasites, a single transmission event is generally assumed, whereas for many, but not all of the agents here, horizontal transmission may also be occurring.

A total of 11 infectious agents demonstrated patterns of seasonal decreases in prevalence in the SW phase concurrent with load truncation in either one or both of the life-history variants; five were exclusive to yearling chinook (*C*.*B*. *cysticola*, *C*. *shasta*, *Loma spp*, *Myxobolus arcticus* and *Renibacterium salmoninarum*) while six displayed similar patterns in both variants. Of those six that had similar patterns in both variants, five reached higher prevalence’s in yearling fish (*P*. *theridion*, *Piscine orthoreovirus (*PRV*)*, salmon gill Chlamydia, *T*. *bryosalmonae* and ENV) while only one reached higher prevalence’s in sub-yearling fish (*P*. *minibicornis*). Thus, while each of these 11 agents is present in both types, they follow different trends in abundance and load. That being said, although we were attempting to not bias the analysis at the outset based on what constitutes a high or low load, it is true that over the temporal period of our study, loads of PRV, salmon gill Chlamidia, and *P*. *theridion* did not reach levels that one would generally consider high where fish may show adverse symptoms of infection [4, 24].

#### Agents displaying truncation exclusive to yearling fish

Three of the five agents that showed truncation only in yearling fish have been linked to disease in juvenile salmon. The other two are linked with adverse effects in salmonids as described briefly below.

1. *Ceratonova shasta (*previously *Ceratomyxa)—*is a myxozoan parasite causing ceratomyxosis, and can result in considerable mortality in juvenile salmonids, including Chinook salmon, and declines in adult returns as well as severe economic impacts on aquaculture [76, 77, 78]. *C*. *shasta* displayed load truncation in yearling fish in the summer-fall period and was completely absent in winter. Despite being FW origin, prevalence actually increased in sub-yearling fish over time. Indeed prevalence was approximately twice as high in sub-yearling fish but no load truncation was observed; this despite an abrupt decline in prevalence between fall and winter.
2. *Loma spp*–is a common gill microsporidium parasite of salmonid fishes with prevalent infections in both wild and captive populations of salmon and trout and disease reported in both freshwater and saltwater phases (reviewed in [79]). The agent causes severe inflammatory lesions associated with ruptured xenomas in the gill tissue [79]. Here, *Loma spp* load truncation was observed in yearling individuals as prevalence declined in fall and disappeared by winter. The agent was present in ocean-type fish at moderate levels.
3. *Renibacterium salmoninarum—*bacteria, the causative agent of bacterial kidney disease (BKD), occurs in salmonid populations worldwide and is endemic to the Salish Sea [80]. In addition to asymptomatic fish, other non-salmonid species can be reservoirs [80] with both vertical and horizontal transmission routes [67, 80]. Interestingly, the agent has also been associated with indirect effects as Chinook salmon challenged with *R*. *salmoninarum* under experimental conditions are more susceptible to predation by other fish predators [81]. Here, the agent demonstrated an abrupt decline from FW to SW. That being said all infected fish were from the same hatchery (Spius Creek) so it is unclear how this is representative for the stock as a whole.
4. *C*.*B*. *cysticola—*bacteria, was recently discovered in Norway, and has been linked to proliferative gill inflammation [68]. High prevalence has been a characteristic of observations across samples in Norway and Ireland [82]. While highly prevalent in juvenile (present study) and adult [17] Chinook salmon, it has also been detected in other species of Pacific salmon in the wild at varying life stages (K. Miller, unpublished data). Here, *C*.*B*. *cysticola* load truncation was observed in winter for both life-history types although the types diverged in summer and fall prevalence (stream type > ocean type). Thereafter, prevalence tended to increase in ocean-type fish, while declined in stream-type fish between fall and winter concurrent with load truncation.
5. *Myxobolus arcticus*—is a FW myxosporean with an alternate oligochaete host found along the North Pacific coast. It infects the central nerve tissues of salmonids for the remainder of the fish’s life [58, 59] and is associated with impaired swim performance [58]. Here, *M*. *arcticus* demonstrated a declining pattern in prevalence and load in stream-type fish in winter, increasing in ocean-type fish in winter despite a presumed FW transmission.

#### Agents with truncation patterns in both life history variants

Six agents demonstrated similar prevalence/load truncation patterns in both life history variants yet five displayed higher maximum prevalences in yearling fish; most are known to cause disease in salmon in the ocean. These are summarized below:

1. *Parvicapsula theridion—*is *a* myxozoan parasite transmitted through sea lice and associated with proliferative gill inflammation (PGI) in salmon in Norway [65, 83]. The parasite develops in cells of the reticuloendothelial system, which are important for normal immune function and is suspected to increase susceptibility of the host to other infectious agents [65]. *P*. *theridion* reached maximum prevalence in the fall for both variants before disappearing from winter samples. This timing of disappearance fits what is known about timing of disease onset for this agent [65].
2. *Tetracapsuloides bryosalmonae—*is a myxozoan parasite transmitted through an alternate bryozoan host in FW and is the causative agent of proliferative kidney disease (PKD) [63, 84]. The disease, characterized by a severe swelling of the kidney induced by a host immune response to the presence of extrasporogonic stages of the parasite, is highly problematic for fish farms and hatcheries, where up to 100% of stock can be infected and mortalities can reach 95%. Here, prevalence of *T*. *bryosalmonae* peaked in fall for both life-history types, but dropped to zero in the winter.
3. *Parvicapsula minibicornis*—is a myxozoan parasite transmitted through an intermediate polychaete host residing in estuarine environments [85]. In severe infection, the parasite causes necrosis within renal tubules and has been associated with pre-mature mortality of adult Sockeye salmon; no studies have been carried out to date on disease potential in juvenile salmon in the marine environment, although the parasite has been associated with increased predation risk in juvenile Sockeye salmon [4]. In our study, *P*. *minibicornis* reached a higher prevalence in sub-yearling than yearling fish, but showed a significant declining pattern for both life-history types in winter, when load truncated was also observed.
4. Piscine orthoreovirus *(*PRV*)—*is an RNA virus in the reovirus family and the causative agent of heart and skeletal muscle inflammation (HSMI) in farmed Atlantic salmon [86], a disease that causes low level mortality on farms (0–20%) but has become a production issue in Norway due to its high prevalence across farms (affecting 100’s of farms per year) [87]. The viral agent and disease has also been observed in farmed Atlantic salmon in Chile [88], and was recently reported by our team on a BC salmon farm [23]. While the disease HSMI has not been documented in *Oncorhynchus spp*., other PRV-related diseases have been observed around the world, most displaying a combination of jaundice, anemia, and heart lesions [88, 89, 90]. However, these diseases have all involved different strains of PRV from that associated with HSMI. PRV was observed at low prevalence in both sub-yearling and yearling salmon, disappearing in winter samples.
5. Erythrocytic necrosis virus (ENV)—is a DNA virus that can cause erythrocytic inclusions and severe anemia in a wide and diverse range of species of marine and anadromous fishes in both the Atlantic and Pacific Oceans [66, 67, 91]. In salmon, ENV infection can result in osmoregulatory difficulties [92]. The virus is presumed to be maintained in populations of marine fish where transmission is thought to be horizontal [66, 92, 93]. Prevalence of ENV increased significantly in the spring in SW then declined in fall and disappeared by winter for both life-history types. Load truncation was observed in fall for yearling fish prior to complete absence in winter for both types.
6. Salmon gill Chlamydia*—*is a bacterial Chlamydia agent recently identified in association with proliferative gill disease in farmed salmon in Europe [94]. Load truncation and declining prevalence was observed in fall for yearling individuals whereas prevalence (and load) dropped to zero by winter in sub-yearling fish.

### Multivariate infectious agent profiles

Salmon sampled in the ocean in our study carried an average co-infection rate of four agents. While these multivariate profiles varied significantly by season and life history type, the effect size was small. This suggests that other factors not considered here are influencing infectious agent profiles in fish. Interestingly, there were no spatial differences detected on the scale of this study (~700 km, mostly within the Salish Sea), suggesting fish are invariant in a seemingly common environment, while profiles shift temporally. Although we observed associations between certain infectious agents, this was a small number; 8 agents in 3 groups. Two infectious agents that share the same intermediate host, namely *P*. *minibicornis* and *C*. *shasta*, displayed the highest level of concordant trends in abundance. However, this association was more prevalent in sub-yearling fish, which carried higher overall prevalences of these agents. Both agents increased in prevalence from spring through fall in SW suggesting that there could be a marine source of infection or that salmon are residing within the vicinity of coastal FW sources longer than presumed. These two agents were also linked with another myxozoan, *T*. *bryosalmonae*, which is transmitted through an alternate FW bryozoan host [63, 85], again consistent with exposure in coastal environments. The second group, consisting of *I*. *hoferii* and *Loma spp*., is associated with a common route of transmission via direct ingestion of infected fish [95]; both were more common in yearling fish, which, as mentioned above, are likely to move to a piscivorous diet earlier in the marine existence than smaller sub-yearling fish [36, 37, 38]. The final group, comprised of ENV, *C*.*B*. *cysticola*, and *M*. *arcticus* are disparate in terms of type of infectious agent and origin. The association among them could simply be an artifact of high prevalence, particularly of *C*.*B*. *cysticola*, or it is possible that they share similar requirements for infection including host immune status, host density, or environmental factors. Fish with ENV may develop severe anemia that may predispose them to other infections [93, 96]. In natural disease outbreaks with significant mortality, ENV is often seen in conjunction with other infections [97] or in association with severe environmental stressors [96]. *C*.*B*. *cysticola* proliferation may benefit from co-infection with other agents [98]. It is also possible that if certain infectious agents are in fact having a disproportionate influence on mortality, given the commonality of multiple co-infections; this will influence the abundance of other infectious agents in non-predictable ways.

Our knowledge of the presence and abundance of suites of infectious agents in wild animal populations has been constrained to date by our ability to easily screen for them; limiting our understanding of what role these might play in survival or much broader questions around the ecological and evolutionary importance of disease processes [4]. The status for all Fraser River CUs has not yet been established [39], but of those that have, a pattern with agent profiles has emerged. Interestingly, the CU’s in decline are of the life-history type showing the highest diversity and prevalence of infectious agents, as well as an epidemiological profile that may, in many cases, be associated with mortality. We hypothesize that due to differences in exposure, the two life-history variants are not impacted by direct effects of disease or secondary cumulative effects in the same way. How this might be manifested is unclear at this point and the purview of future work.

There are limitations and assumptions to our approach and current data set beyond sample sizes. Due to the difficulty of obtaining consistently sufficient sample sizes to contrast patterns among co-migrating stocks, we did not perform stock-specific analyses, but the current analysis points to stock as a potential important consideration. The assumption is made that we have representatively sampled the Chinook salmon stock(s) appropriately through time. At present, samples were obtained opportunistically from current DFO sampling programs. While these coastal surveys, spaced 2–4 months apart, generally replicate spatial coverage between seasons, they are in the pelagic zone (typically greater than 30m) not in nearshore or estuarine habitats. Therefore they do not cover the entire range of habitats occupied by juvenile fish during their outmigration from fresh water to salt water. Thus, these specific samples may not necessarily be at the spatial (habitats) or temporal scales (sampling interval) at which particular infectious agent dynamics potentially operate. The program is in the process of collating within-river and nearshore samples taken on shorter time intervals (weeks) in other years that can be assessed independently to validate findings herein. The advantage of the present dataset, however, is the replication of consistent sampling over time. In this study, our perspective of FW pattern is at presently restricted to hatchery samples. However, this is ameliorated in part by the fact that SW samples in spring were likely only resident for a period of days to weeks, likely integrating a FW “signal” in infectious agent profiles. This is also true for SW summer samples in sub-yearling fish given that they tended to be dominated by South Thompson individuals. Clearer differentiation will be attained by the analysis of additional samples obtained from the exit point in the Fraser.

Our presentation focused on particular agents that displayed interesting patterns of prevalence and load consistent with both agent activity (replication) and loss. Ultimately our definition of which infectious agents fall out as being most relevant will be fluid as our knowledge expands both through the analysis of additional stocks, species and years as well as directed studies that integrate quantitative infectious agent monitoring with disease and impact assessments at the molecular (gene expression profiling), cellular (histopathology), and organismal (holding studies, challenge studies, tracking studies, predation studies) levels. Granted, the interpretation of some of these patterns will require further exploration; there are alternative plausible explanations. Agent patterns could be further influenced by 1) longitudinal samples not representing the same fish “population” through time, 2) transmission of the particular infectious agent in alternate environments than anticipated, or 3) fish are using estuarine areas more frequently than thought. One of the analytic challenges is interpreting these in a co-infection, multivariate context. Increased sample sizes and trends in different species and stocks will provide greater insight. Patterns may be revealed through both a broader, more thorough contrast between greater number of stocks and/or species, coastal regions and temporal scales as well as stock-specific fine scale pattern changes.

## Supporting information

S1 Table TaqMan assays for 45 infectious agents and one host reference gene run on juvenile Chinook salmon mixed-tissue samples using the Fluidigm Biomark HT-qRT-PCR platform (DFO Pacific Biological Station, Nanaimo, BC) and their design origin. (DOCX)

S2 Table Known or postulated transmission environment, infectious agent class and type for specific agents found at a mean prevalence >1% in Fraser River Chinook salmon. (DOCX)

S3 Table Results of least-square means analysis testing season and life-history effects on infectious agent prevalence in juvenile Fraser River Chinook salmon. (DOCX)

S4 Table Prevalence and load trends of infectious agents detected in juvenile Fraser River Chinook salmon. (DOCX)
