# Supplementary material for: Distinct seasonal infectious agent profiles in life-history variants of juvenile Fraser River Chinook salmon: An application of high-throughput genomic screening
Source: PLoS One. 2018 Apr 19;13(4):e0195472. doi: 10.1371/journal.pone.0195472 (PMC5908190; doi:10.1371/journal.pone.0195472)
Supplement: S1 Table — (DOCX) [file pone.0195472.s001.docx]

**Supplementary material**

**S1 Table. TaqMan assays for 45 infectious agents and one host reference gene run on juvenile Chinook salmon mixed-tissue samples using the Fluidigm Biomark HT-qRT-PCR platform (DFO Pacific Biological Station, Nanaimo, BC) and their design origin.** MGL refers to an in-house assay design. Assays with an asterisk (*) were added to replace poorly performing assays (isav7, vhsv, pmcv) after the initial platform evaluation. ^†^ determined to be stably expressed across species, tissues and migration states in Pacific salmon and is therefore used as a housekeeping gene.

|  | Full Name | Assay Abbreviation | Assay Reference | Forward (5’-3’)  Reverse (5’-3’)  Probe (FAM-5’-3’-MGB) |  |
| --- | --- | --- | --- | --- | --- |
| **Bacteria** | *Aeromonas hydrophila* | ae.hyd | [1] | F: ACCGCTGCTCATTACTCTGATG  R: CCAACCCAGACGGGAAGAA  P: TGATGGTGAGCTGGTTG |  |
|  | *Aeromonas salmonicida* | ae.sal | modification of [2] | F: TAAAGCACTGTCTGTTACC  R: GCTACTTCACCCTGATTGG  P: ACATCAGCAGGCTTCAGAGTCACTG |  |
|  | *Candidatus Branchiomonas cysticola* | c.b.cys | [3] | F: AATACATCGGAACGTGTCTAGTG  R: GCCATCAGCCGCTCATGTG  P: CTCGGTCCCAGGCTTTCCTCTCCCA |  |
|  | *Flavobacterium psychrophilum* | fl.psy | [4] | F: GATCCTTATTCTCACAGTACCGTCAA  R: TGTAAACTGCTTTTGCACAGGAA  P: AAACACTCGGTCGTGACC |  |
|  | Gill chlamydia | sch | [4] | F: GGGTAGCCCGATATCTTCAAAGT  R: CCCATGAGCCGCTCTCTCT  P: TCCTTCGGGACCTTAC |  |
|  | *Piscichlamydia salmonis* | pch.sal | [5] | F: TCACCCCCAGGCTGCTT  R: GAATTCCATTTCCCCCTCTTG  P: CAAAACTGCTAGACTAGAGT |  |
|  | *Piscirickettsia salmonis* | pisck.sal | [6] | F: TCTGGGAAGTGTGGCGATAGA  R: TCCCGACCTACTCTTGTTTCATC  P: TGATAGCCCCGTACACGAAACGGCATA |  |
|  | *Renibacterium salmoninarum* | re.sal | [7] | F: CAACAGGGTGGTTATTCTGCTTTC  R: CTATAAGAGCCACCAGCTGCAA  P: CTCCAGCGCCGCAGGAGGAC |  |
|  | Rickettsia-like organism | rlo | [8] | F: GGCTCAACCCAAGAACTGCTT  R: GTGCAACAGCGTCAGTGACT  P: CCCAGATAACCGCCTTCGCCTCCG |  |
|  | *Vibrio anguillarum* | vi.ang | MGL | F: CCGTCATGCTATCTAGAGATGTATTTGA  R: CCATACGCAGCCAAAAATCA  P: TCATTTCGACGAGCGTCTTGTTCAGC |  |
|  | *Vibrio salmonicida* | vi.sal | MGL | F: GTGTGATGACCGTTCCATATTT  R: GCTATTGTCATCACTCTGTTTCTT  P: TCGCTTCATGTTGTGTAATTAGGAGCGA |  |
|  |  |  |  |  | |
| **Parasites** | *Ceratomyxa shasta* | ce.sha | [9] | F: CCAGCTTGAGATTAGCTCGGTAA  R: CCCCGGAACCCGAAAG  P: CGAGCCAAGTTGGTCTCTCCGTGAAAAC |  |
|  | *Cryptobia salmositica* | cr.sal | MGL | F: TCAGTGCCTTTCAGGACATC  R: GAGGCATCCACTCCAATAGAC  P: AGGAGGACATGGCAGCCTTTGTAT |  |
|  | *Dermocystidium salmonis* | de.sal | MGL | F: CAGCCAATCCTTTCGCTTCT  R: GACGGACGCACACCACAGT  P: AAGCGGCGTGTGCC |  |
|  | *Facilispora margolisi* | fa.mar | MGL | F: AGGAAGGAGCACGCAAGAAC  R: CGCGTGCAGCCCAGTAC  P: TCAGTGATGCCCTCAGA |  |
|  | *Gyrodactylus salaris* | gy.sal | [10] | F: CGATCGTCACTCGGAATCG  R: GGTGGCGCACCTATTCTACA  P: TCTTATTAACCAGTTCTGC |  |
|  | *Ichthyophonus hoferi* | ic.hof | [11] | F: GTCTGTACTGGTACGGCAGTTTC  R: TCCCGAACTCAGTAGACACTCAA  P: TAAGAGCACCCACTGCCTTCGAGAAGA |  |
|  | *Ichthyophthirius multifiliis* | ic.mul | MGL | F: AAATGGGCATACGTTTGCAAA  R: AACCTGCCTGAAACACTCTAATTTTT  P: ACTCGGCCTTCACTGGTTCGACTTGG |  |
|  | *Kudoa thyrsites* | ku.thy | [12] | F: TGGCGGCCAAATCTAGGTT  R: GACCGCACACAAGAAGTTAATCC  P: TATCGCGAGAGCCGC |  |
|  | *Loma spp.* | lo.sal | MGL | F: GGAGTCGCAGCGAAGATAGC  R: CTTTTCCTCCCTTTACTCATATGCTT  P: GCCTGAAATCACGAGAGTGAGACTACCC |  |
|  | *Myxobolus arcticus* | my.arc | MGL | F: TGGTAGATACTGAATATCCGGGTTT  R: AACTGCGCGGTCAAAGTTG  P:CGTTGATTGTGAGGTTGG |  |
|  | *Myxobolus cerebralis* | my.cer | [13] | F: GCCATTGAATTTGACTTTGGATTA  R: ACCATTCATGTAAGCCCGAACT  P: TCGAAGCCTTGACCATCTTTTGGCC |  |
|  | *Myxobolus insidiosus* | my.ins | MGL | F: CCAATTTGGGAGCGTCAAA  R: CGATCGGCAAAGTTATCTAGATTCA  P: CTCTCAAGGCATTTAT |  |
|  | *Nanophyetus salmincola* | na.sal | MGL | F: GATCTGCATTTGGTTCTGTAACA  R: CCAACGCCACAATGATAGCTATAC  P: TGAGGCGTGTTTTATG |  |
|  | *Neoparamoeba perurans* | ne.per | [14] | F: GTTCTTTCGGGAGCTGGGAG  R: GAACTATCGCCGGCACAAAAG  P: CAATGCCATTCTTTTCGGA |  |
|  | *Nucleospora salmonis* | nu.sal | [15] | F: GCCGCAGATCATTACTAAAAACCT  R: CGATCGCCGCATCTAAACA  P: CCCCGCGCATCCAGAAATACGC |  |
|  | *Paranucleospora theridion* (syn. *Desmozoon lepeophtherii*) | pa.ther | [16] | F: CGGACAGGGAGCATGGTATAG  R: GGTCCAGGTTGGGTCTTGAG  P: TTGGCGAAGAATGAAA |  |
|  | *Parvicapsula kabatai* | pa.kab | MGL | F: CGACCATCTGCACGGTACTG  R: ACACCACAACTCTGCCTTCCA  P:CTTCGGGTAGGTCCGG |  |
|  | *Parvicapsula minibicornis* | pa.min | [17] | F: AATAGTTGTTTGTCGTGCACTCTGT  R: CCGATAGGCTATCCAGTACCTAGTAAG  P: TGTCCACCTAGTAAGGC |  |
|  | *Parvicapsula pseudobranchicola* | pa.pse | [18] | F: CAGCTCCAGTAGTGTATTTCA  R: TTGAGCACTCTGCTTTATTCAA  P: CGTATTGCTGTCTTTGACATGCAGT |  |
|  | *Sphaerothecum destruens* | sp.des | MGL | F: GGGTATCCTTCCTCTCGAAATTG  R: CCCAAACTCGACGCACACT  P: CGTGTGCGCTTAAT |  |
|  | *Spironucleus salmonicida* | sp.sal | MGL | F: GCAGCCGCGGTAATTCC  R: CGAACTTTTTAACTGCAGCAACA  P: ACACGGAGAGTATTCT |  |
|  | *Tetracapsuloides bryosalmonae* | te.bry | [19] | F: GCGAGATTTGTTGCATTTAAAAAG  R: GCACATGCAGTGTCCAATCG  P: CAAAATTGTGGAACCGTCCGACTACGA |  |
|  |  |  |  |  |  |
| **Viruses** | Atlantic salmon paramyxovirus | aspv | [5] | F: CCCATATTAGCAAATGAGCTCTATCTT  R: CGTTAAGGAACTCATCATTGAGCTT  P: AGCCCTTTTGTTCTGC |  |
|  | Infectious hematopoietic necrosis virus | ihnv | [20] | F: AGAGCCAAGGCACTGTGCG  R: TTCTTTGCGGCTTGGTTGA  P: TGAGACTGAGCGGGACA |  |
|  | Infectious pancreatic necrosis virus | ipnv | [21] | F: GCAACTTACTTGAGATCCATTATGCT  R:AGACCTCTAAGTTGTATGACGAGGTCTCT  P: CGAGAATGGGCCAGCAAGCA |  |
|  | Infectious salmon anemia virus | isav7 | [22] | F: CAGGGTTGTATCCATGGTTGAAATG  R: GTCCAGCCCTAAGCTCAACTC  P: CTCTCTCATTGTGATCCC |  |
|  | Infectious salmon anemia virus | isav8* | [23] | F: TGGGCAATGGTGTATGGTATGA  R: GAAGTCGATGAACTGCAGCGA  P: CAGGATGCAGATGTATGC |  |
|  | Pacific salmon parvovirus | pspv | MGL | F: CCCTCAGGCTCCGATTTTTAT  R: CGAAGACAACATGGAGGTGACA  P: CAATTGGAGGCAACTGTA |  |
|  | Piscine myocarditis virus (CMS) | pmcv | [24] | F: TTCCAAACAATTCGAGAAGCG  R: ACCTGCCATTTTCCCCTCTT  P: CCGGGTAAAGTATTTGCGTC |  |
|  | Piscine myocarditis virus (CMS) | pmcv1* | [25] | F: AGGGAACAGGAGGAAGCAGAA  R: CGTAATCCGACATCATTTTGTGA  P: TGGTGGAGCGTTCAA |  |
|  | Piscine reovirus (HSMI) | prv | [26] | F: TGCTAACACTCCAGGAGTCATTG  R: TGAATCCGCTGCAGATGAGTA  P: CGCCGGTAGCTCT |  |
|  | Salmon alphavirus 1, 2, and 3 (PD/SD/HSS) | sav | [27] | F: CCGGCCCTGAACCAGTT  R: GTAGCCAAGTGGGAGAAAGCT  P: TCGAAGTGGTGGCCAG |  |
|  | Salmonid herpesvirus/ *Oncorhynchus masou* herpes virus | omv | MGL | F: GCCTGGACCACAATCTCAATG R: CGAGACAGTGTGGCAAGACAAC P: CCAACAGGATGGTCATTA |  |
|  | Viral encephalopathy and retinopathy virus | ver | [28] | F: TTCCAGCGATACGCTGTTGA  R: CACCGCCCGTGTTTGC  P: AAATTCAGCCAATGTGCCCC |  |
|  | Viral erythrocytic necrosis | env | [29] | F: CGTAGGGCCCCAATAGTTTCT  R: GGAGGAAATGCAGACAAGATTTG  P: TCTTGCCGTTATTTCCAGCACCCG |  |
|  | Viral hemorrhagic septicemia virus | vhsv | [30] | F: ATGAGGCAGGTGTCGGAGG  R: TGTAGTAGGACTCTCCCAGCATCC  P: TACGCCATCATGATGAGT |  |
|  | Viral hemorrhagic septicemia virus | vhsv1* | [31] | F: AACTCGCAGGATGTGTGCGTCC  R: TCTGCGATCTCAGTCAGGATGAA  P: TAGAGGGCCTTGGTGATCTTCTG |  |
|  |  |  |  |  |  |
| **Housekeeping gene** | 78d16.1^†^ |  | MGL | F: GTCAAGACTGGAGGCTCAGAG  R: GATCAAGCCCCAGAAGTGTTTG  P: AAGGTGATTCCCTCGCCGTCCGA |  |

1. Lee D-Y, Shannon K, Beaudette LA. Detection of bacterial pathogens in municipal wastewater using an oligonucleotide microarray and real-time quantitative PCR. J Microbiol Methods. 2006;65:453–467.
2. Keeling SE, Brosnahan CL, Johnston C, Wallis R, Gudkovs N. Development and validation of a real-time PCR assay for the detection of *Aeromonas salmonicida*. J Fish Dis. 2013;1–9.
3. Mitchell SO, Steinum TM, Toenshoff ER, Kvellestad A, Falk K, Horn M, Colquhoun DJ. Candidatus *Branchiomonas cysticola* is a common agent of epitheliocysts in seawater-farmed Atlantic salmon *Salmo salar* in Norway and Ireland. Dis Aquat Organ. 2013;03(1):35-43. doi: 10.3354/dao02563.
4. Duesund, H., Nylund S, Watanabe K, Ottem KF, Nylund A. Characterization of a VHS virus genotype III isolated from rainbow trout (*Oncorhynchus mykiss*) at a marine site of the west coast of Norway. Virol J. 2010;7–19.
5. Nylund A, Watanabe K, Nylund S, Karlsen M, Sæther PA, Arnesen CE, Karlsbakk E. Morphogenesis of salmonid gill poxvirus associated with proliferative gill disease in farmed Atlantic salmon (*Salmo salar*) in Norway. Arch Virol. 2008;153:1299–1309.
6. Corbeil S, McColl KA, Crane MSJ. Development of a TaqMan quantitative PCR assay for the identification of *Piscirickettsia salmonis*. Bull Eur Assn Fish P. 2003;23(3):95-101.
7. Powell M, Overturf K, Hogge C, Johnson K. Detection of *Renibacterium salmoninarum* in Chinook salmon, *Oncorhynchus tshawytscha* (Walbaum), using quantitative PCR. J Fish Dis. 2005;28:615–622.
8. Lloyd, SJ, LaPatra SE, Snekvik KR, Cain KD, Call DR. Quantitative PCR demonstrates a positive correlation between a Rickettsia-like organism and severity of strawberry disease lesions in rainbow trout, *Oncorhynchus mykiss* (Walbaum). J Fish Dis. 2011;34:701–709.
9. Hallett SL, Bartholomew JL. Application of a real-time PCR assay to detect and quantify the myxozoan parasite *Ceratomyxa shasta* in river water samples. Dis Aquat Organ. 2006;71:109–18.
10. Collins CM, Kerr R, Mcintosh R, M. Development of a real-time PCR assay for the identification of *Gyrodactylus* parasites infecting salmonids in northern Europe. Dis Aquat Organ. 2010;90:135–42.
11. White VC, Morado JF, Crosson LM, Vadopalas B,Friedman CS. Development and validation of a quantitative PCR assay for *Ichthyophonus* spp. Dis Aquat Organ. 2013; 104:69-81.
12. Funk VA, Raap M, Sojonky K, Jones S, Robinson J, Falkenberg C, et al. Development and validation of an RNA- and DNA- based quantitative PCR assay for determination of *Kudoa thyrsites* infection levels in Atlantic salmon *Salmo salar*. Dis Aquat Organ. 2007;75:239–49.
13. Kelley GO, Zagmutt-Vergara FJ, Leutenegger CM, Adkison MA, Rava DV, Hedrick RP. Identification of a serine protease gene expressed by *Myxobolus cerebralis* during development in rainbow trout *Oncorhynchus mykiss*. Dis Aquat Organ. 2004;59: 235-248.
14. Fringuelli E, Gordon AW, Rodger H, Welsh MD, Graham DA. Detection of *Neoparamoeba perurans* by duplex quantitative Taqman real-time PCR in formalin-fixed, paraffin-embedded Atlantic salmonid gill tissues. J Fish Dis. 2012;711–24.
15. Foltz JR, Plant KP, Overturf K, Clemens K, Powell MS. Detection of Nucleospora salmonis in steelhead trout , *Oncorhynchus mykiss* (Walbaum), using quantitative polymerase chain reaction (qPCR). J Fish Biol. 2009;32:551–5.
16. Nylund S, Nylund A, Watanabe K, Arnesen CE, Karlsbakk E. *Paranucleospora theridion* n. gen., n. sp. (Microsporidia, Enterocytozoonidae) with a life cycle in the salmon louse (*Lepeophtheirus salmonis*, Copepoda) and Atlantic salmon (*Salmo salar*). J Eukaryot Microbiol. 2010;57:95–114.
17. Hallett SL, Bartholomew JL. Development and application of a duplex QPCR for river water samples to monitor the myxozoan parasite *Parvicapsula minibicornis*. Dis Aquat Organ. 2009;86:39–50.
18. Jorgensen A, Nylund A, Nikolaisen V, Alexandersen S, Karlsbakk E. Real-time PCR detection of *Parvicapsula pseudobranchicola* (Myxozoa :Myxosporea) in wild salmonids in Norway. J Fish Dis. 2011;365–71.
19. Bettge K, Wahli T, Segner H, Schmidt-Posthaus H. Proliferative kidney disease in rainbow trout : time- and temperature-related renal pathology and parasite distribution. Dis Aquat Organ. 2009;83:67–76.
20. Purcell MK, Thompson RL, Garver KA, Hawley LM, Batts WN, Sprague L, Sampson C, JR Winton. Universal reverse-transcriptase real-time PCR for infectious hematopoietic necrosis virus (IHNV). Dis Aquat Organ. 2013;106:103-115.
21. Clouthier S, T. Schroeder C. McClure, M. Lindsay, S. Khatkar, C. Collette-Belliveau, L. Gaudet, E. Johnsen, J. Allen, A. Zetner, E. Anderson. 2014. Development and diagnostic validation of a reverse transcription quantitative PCR (RT-qPCR) assay for detection of infectious pancreatic necrosis virus (IPNV). 7th International Symposium on Aquatic Animal Health ISAAH, Portland USA, August 31st-September 4th, 2014.
22. Snow M, McKay P, McBeath AJ, Black J, Doig F, Kerr R et al. Development, application and validation of a Taqman real-time RT-PCR assay for the detection of infectious · salmon anaemia virus (ISAV) in Atlantic salmon (Salmo salar). Dev Biol(Basel) 2006;126:133-45.
23. LeBlanc, F., M. Laflamme, and N. Gagné. Genetic markers of the immune response of Atlantic salmon (Salmo salar) to infectious salmon anemia virus (ISAV). Fish Shellfish Immunol. 2010;29:217-232, dio: 10.1016/j.fsi.2010.03.007
24. Løvoll M, Wiik-Nielsen J, Grove S, Wiik-Nielsen CR, Kristoffersen AB, et al. A novel totivirus and piscine reovirus (PRV) in Atlantic salmon (*Salmo salar*) with cardiomyopathy syndrome (CMS). Virology Journal 2010;7:309.
25. Wiik-Nielsen J, Alarcon M, Fineid B, Rode M, Haugland Ø. Genetic variation in Norwegian piscine myocarditis virus in Atlantic salmon, *Salmo salar* L. J Fish Dis. 2013;36: 129-139.
26. Wiik-Nielsen CR, Ski P-MR, Aunsmo A, Løvoll M. Prevalence of viral RNA from piscine reovirus and piscine myocarditis virus in Atlantic salmon, *Salmo salar* L broodfish and progeny. J Fish Dis. 2011;35:169–171.
27. Andersen L, Bratland A, Hodneland K, Nylund A. Tissue tropism of salmonid alphaviruses (subtypes SAV1 and SAV3) in experimentally challenged Atlantic salmon (*Salmo salar* L.). Arch Virol. 2007;152:1871–83.
28. Korsnes K, Devold M, Nerland AH, Nylund A. Viral encephalopathy and retinopathy (VER) in Atlantic salmon *Salmo salar* after intraperitoneal challenge with a nodavirus from Atlantic halibut *Hippoglossus hippoglossus*. Dis Aquat Organ. 2005;68:7-15.
29. Purcell MK, Pearman-Gillman S, Thompson RL, Gregg JL, Hart LM, Winton JR, et al. Identification of the major capsid protein of erythrocytic necrosis virus (ENV) and development of quantitative real-time PCR assays for quantification of ENV DNA. J Vet Diagn Invest. 2016;28:382-391.
30. Garver KA, Hawley LM, Mcclure CA, Schroeder T, Aldous S, Doig F, et al. Development and validation of a reverse transcription quantitative PCR for universal detection of viral hemorrhagic septicemia virus. Dis Aquat Organ. 2011;95:97–112.
31. Jonstrup SP, Kahns S, Skall HF, Boutrup TS, Olesen NJ. Development and validation of a novel Taqman-based real-time RT-PCR assay suitable for demonstrating freedom from viral haemorrhagic septicaemia virus. J Fish Dis. 2013;9–23.
