# Supplementary material for: Distinct seasonal infectious agent profiles in life-history variants of juvenile Fraser River Chinook salmon: An application of high-throughput genomic screening
Source: PLoS One. 2018 Apr 19;13(4):e0195472. doi: 10.1371/journal.pone.0195472 (PMC5908190; doi:10.1371/journal.pone.0195472)
Supplement: S2 Table — (DOCX) [file pone.0195472.s002.docx]

**S2 Table. Known or postulated transmission environment, infectious agent class and type for specific agents found at a mean prevalence >1% in Fraser River Chinook salmon.**

| transmission | class | type | Infectious agent | abbreviation |
| --- | --- | --- | --- | --- |
| FW | bacteria | bacteria | *Flavobacteria psychrophilum* | fl.psy |
|  | parasite | myxozoan | *Ceratonova shasta* | ce.sha |
|  | parasite | myxozoan | *Myxobolus arcticus* | my.arc |
|  | parasite | myxozoan | *Parvicapsula minibicornis* | pa.min |
|  | parasite | protozoan | *Ichthyophthirius multifiliis* | ic.mul |
|  |  |  |  |  |
| FW & SW | bacteria | bacteria | *Renibacteria salmoninarum* | re.sal |
|  | bacteria | bacteria | *Rickettsia-like organism* | rlo |
|  | parasite | choanoflagellate | *Sphaerothecum destructuens* | sp.des |
|  | parasite | microsporidium | *Loma spp*** | lo.spp |
|  | parasite | myxozoan | *Tetracapsuloides bryosalmonae* | te.bry |
|  | parasite | protozoan | *Ichthyophonus hoferi* | ic.hof |
|  | virus | virus | *Piscine reovirus* | prv |
|  | virus | virus | *Viral hemorrhagic septicemia virus* | vhsv |
|  |  |  |  |  |
| SW | bacteria | bacteria | *Candidatus Branchiomonas cysticola* | c.b.cys |
|  | bacteria | bacteria | *Salmon Gill chlamydia* | sch |
|  | parasite | microsporidium | *Facilispora margolisi* | fa.mar |
|  | parasite | microsporidium | *Paranucleospora theridion* | pa.ther |
|  | parasite | myxozoan | *Kudoa thyrsites* | ku.thy |
|  | parasite | myxozoan | *Parvicapsula kabatai* | pa.kab |
|  | parasite | myxozoan | *Parvicapsula pseudobranchicola* | pa.pse |
|  | virus | virus | *Erythrocytic necrosis virus* | env |

Transmission environment is freshwater (FW), saltwater (SW) or both. Infectious agent class is bacteria, parasite or virus. ** the lo.spp assay is genus, not species-specific.
