# Supplementary material for: Distinct seasonal infectious agent profiles in life-history variants of juvenile Fraser River Chinook salmon: An application of high-throughput genomic screening
Source: PLoS One. 2018 Apr 19;13(4):e0195472. doi: 10.1371/journal.pone.0195472 (PMC5908190; doi:10.1371/journal.pone.0195472)
Supplement: S3 Table — (DOCX) [file pone.0195472.s003.docx]

**S3 Table. Results of least-square means analysis testing season and life-history effects on infectious agent prevalence in juvenile Fraser River Chinook salmon.**

|  |  | Df | Sum SQ | Mean Sq | F | P | BH adjP |
| --- | --- | --- | --- | --- | --- | --- | --- |
| c.b.cys | season | 4 | 7.15 | 1.788 | 11.510 | 0.000 | 0.000 |
|  | lifehistory | 1 | 2.57 | 2.572 | 16.562 | 0.000 | 0.000 |
|  | season:lifehistory | 4 | 2.45 | 0.612 | 3.939 | 0.004 | 0.010 |
|  | Residuals | 645 | 100.17 | 0.155 |  |  |  |
|  |  |  |  |  |  |  |  |
| ce.sha | season | 4 | 14.91 | 3.727 | 21.444 | 0.000 | 0.000 |
|  | lifehistory | 1 | 1.96 | 1.964 | 11.303 | 0.001 | 0.003 |
|  | season:lifehistory | 4 | 2.02 | 0.504 | 2.898 | 0.021 | 0.052 |
|  | Residuals | 645 | 112.10 | 0.174 |  |  |  |
|  |  |  |  |  |  |  |  |
| env | season | 4 | 13.73 | 3.432 | 22.412 | 0.000 | 0.000 |
|  | lifehistory | 1 | 0.01 | 0.009 | 0.061 | 0.805 | 0.848 |
|  | season:lifehistory | 4 | 0.40 | 0.099 | 0.649 | 0.628 | 0.784 |
|  | Residuals | 645 | 98.77 | 0.153 |  |  |  |
|  |  |  |  |  |  |  |  |
| fa.mar | season | 4 | 0.17 | 0.043 | 3.195 | 0.013 | 0.034 |
|  | lifehistory | 1 | 0.03 | 0.030 | 2.260 | 0.133 | 0.227 |
|  | season:lifehistory | 4 | 0.05 | 0.014 | 1.016 | 0.398 | 0.558 |
|  | Residuals | 645 | 8.62 | 0.013 |  |  |  |
|  |  |  |  |  |  |  |  |
| fl.psy | season | 4 | 0.26 | 0.065 | 4.471 | 0.001 | 0.005 |
|  | lifehistory | 1 | 0.00 | 0.002 | 0.148 | 0.700 | 0.784 |
|  | season:lifehistory | 4 | 0.13 | 0.033 | 2.277 | 0.060 | 0.122 |
|  | Residuals | 645 | 9.45 | 0.015 |  |  |  |
|  |  |  |  |  |  |  |  |
| ic.hof | season | 4 | 1.07 | 0.269 | 2.126 | 0.076 | 0.150 |
|  | lifehistory | 1 | 0.30 | 0.302 | 2.389 | 0.123 | 0.221 |
|  | season:lifehistory | 4 | 1.18 | 0.295 | 2.333 | 0.054 | 0.118 |
|  | Residuals | 645 | 81.48 | 0.126 |  |  |  |
|  |  |  |  |  |  |  |  |
| ic.mul | season | 4 | 0.06 | 0.015 | 1.641 | 0.162 | 0.262 |
|  | lifehistory | 1 | 0.01 | 0.009 | 1.038 | 0.309 | 0.444 |
|  | season:lifehistory | 4 | 0.02 | 0.005 | 0.562 | 0.691 | 0.784 |
|  | Residuals | 645 | 5.86 | 0.009 |  |  |  |
|  |  |  |  |  |  |  |  |
| ku.thy | season | 4 | 0.31 | 0.078 | 1.796 | 0.128 | 0.224 |
|  | lifehistory | 1 | 0.01 | 0.007 | 0.156 | 0.693 | 0.784 |
|  | season:lifehistory | 4 | 0.22 | 0.054 | 1.244 | 0.291 | 0.436 |
|  | Residuals | 645 | 28.09 | 0.044 |  |  |  |
|  |  |  |  |  |  |  |  |
| lo.sal | season | 4 | 4.11 | 1.027 | 5.153 | 0.000 | 0.002 |
|  | lifehistory | 1 | 0.56 | 0.563 | 2.827 | 0.093 | 0.178 |
|  | season:lifehistory | 4 | 0.43 | 0.108 | 0.541 | 0.706 | 0.784 |
|  | Residuals | 645 | 128.51 | 0.199 |  |  |  |
|  |  |  |  |  |  |  |  |
| my.arc | season | 4 | 15.76 | 3.940 | 18.995 | 0.000 | 0.000 |
|  | lifehistory | 1 | 1.96 | 1.958 | 9.437 | 0.002 | 0.007 |
|  | season:lifehistory | 4 | 2.35 | 0.588 | 2.833 | 0.024 | 0.056 |
|  | Residuals | 645 | 133.79 | 0.207 |  |  |  |
|  |  |  |  |  |  |  |  |
| pa.kab | season | 4 | 0.65 | 0.163 | 4.692 | 0.001 | 0.004 |
|  | lifehistory | 1 | 0.00 | 0.000 | 0.011 | 0.916 | 0.946 |
|  | season:lifehistory | 4 | 0.08 | 0.020 | 0.566 | 0.687 | 0.784 |
|  | Residuals | 645 | 22.39 | 0.035 |  |  |  |
|  |  |  |  |  |  |  |  |
| pa.min | season | 4 | 47.96 | 11.991 | 68.504 | 0.000 | 0.000 |
|  | lifehistory | 1 | 1.67 | 1.671 | 9.549 | 0.002 | 0.007 |
|  | season:lifehistory | 4 | 0.90 | 0.224 | 1.279 | 0.277 | 0.425 |
|  | Residuals | 645 | 112.90 | 0.175 |  |  |  |
|  |  |  |  |  |  |  |  |
| pa.pse | season | 4 | 8.17 | 2.043 | 12.459 | 0.000 | 0.000 |
|  | lifehistory | 1 | 1.93 | 1.926 | 11.745 | 0.001 | 0.003 |
|  | season:lifehistory | 4 | 0.87 | 0.217 | 1.321 | 0.261 | 0.411 |
|  | Residuals | 645 | 105.76 | 0.164 |  |  |  |
|  |  |  |  |  |  |  |  |
| pa.ther | season | 4 | 17.41 | 4.352 | 21.901 | 0.000 | 0.000 |
|  | lifehistory | 1 | 1.64 | 1.644 | 8.272 | 0.004 | 0.012 |
|  | season:lifehistory | 4 | 2.31 | 0.578 | 2.906 | 0.021 | 0.052 |
|  | Residuals | 645 | 128.17 | 0.199 |  |  |  |
|  |  |  |  |  |  |  |  |
| prv | season | 4 | 0.22 | 0.056 | 4.180 | 0.002 | 0.007 |
|  | lifehistory | 1 | 0.00 | 0.005 | 0.340 | 0.560 | 0.764 |
|  | season:lifehistory | 4 | 0.03 | 0.007 | 0.515 | 0.725 | 0.787 |
|  | Residuals | 645 | 8.62 | 0.013 |  |  |  |
|  |  |  |  |  |  |  |  |
| re.sal | season | 4 | 1.52 | 0.381 | 17.867 | 0.000 | 0.000 |
|  | lifehistory | 1 | 1.50 | 1.502 | 70.447 | 0.000 | 0.000 |
|  | season:lifehistory | 4 | 4.48 | 1.121 | 52.572 | 0.000 | 0.000 |
|  | Residuals | 645 | 13.75 | 0.021 |  |  |  |
|  |  |  |  |  |  |  |  |
| rlo | season | 4 | 0.50 | 0.124 | 2.272 | 0.060 | 0.122 |
|  | lifehistory | 1 | 0.06 | 0.056 | 1.033 | 0.310 | 0.444 |
|  | season:lifehistory | 4 | 0.12 | 0.030 | 0.547 | 0.701 | 0.784 |
|  | Residuals | 645 | 35.13 | 0.054 |  |  |  |
|  |  |  |  |  |  |  |  |
| sch | season | 4 | 2.00 | 0.501 | 6.175 | 0.000 | 0.000 |
|  | lifehistory | 1 | 0.01 | 0.005 | 0.059 | 0.808 | 0.848 |
|  | season:lifehistory | 4 | 0.20 | 0.049 | 0.602 | 0.662 | 0.784 |
|  | Residuals | 645 | 52.30 | 0.081 |  |  |  |
|  |  |  |  |  |  |  |  |
| sp.des | season | 4 | 0.10 | 0.025 | 1.869 | 0.114 | 0.211 |
|  | lifehistory | 1 | 0.00 | 0.004 | 0.286 | 0.593 | 0.779 |
|  | season:lifehistory | 4 | 0.01 | 0.003 | 0.208 | 0.934 | 0.949 |
|  | Residuals | 645 | 8.76 | 0.014 |  |  |  |
|  |  |  |  |  |  |  |  |
| te.bry | season | 4 | 6.85 | 1.711 | 15.555 | 0.000 | 0.000 |
|  | lifehistory | 1 | 0.23 | 0.231 | 2.098 | 0.148 | 0.245 |
|  | season:lifehistory | 4 | 0.32 | 0.081 | 0.732 | 0.570 | 0.764 |
|  | Residuals | 645 | 70.96 | 0.110 |  |  |  |
|  |  |  |  |  |  |  |  |
| vhsv | season | 4 | 0.11 | 0.028 | 2.353 | 0.053 | 0.118 |
|  | lifehistory | 1 | 0.00 | 0.002 | 0.139 | 0.710 | 0.784 |
|  | season:lifehistory | 4 | 0.01 | 0.002 | 0.162 | 0.958 | 0.958 |
|  | Residuals | 645 | 7.78 | 0.012 |  |  |  |

See S1 Table for full infectious agent names. P values were adjusted for multiple comparisons using the Benjamini and Hochberg method [47]. * *Paranucleospora theridion* has also been called *Desmozoon lepeophtherii*
