# Supplementary material for: Distinct seasonal infectious agent profiles in life-history variants of juvenile Fraser River Chinook salmon: An application of high-throughput genomic screening
Source: PLoS One. 2018 Apr 19;13(4):e0195472. doi: 10.1371/journal.pone.0195472 (PMC5908190; doi:10.1371/journal.pone.0195472)
Supplement: S4 Table — (DOCX) [file pone.0195472.s004.docx]

**S4 Table: Prevalence and load trends of infectious agents detected in juvenile Fraser River Chinook salmon.** See main text Table 1 for full infectious agent names. Transmission environment noted. Seasonal prevalences are differentiated by life-history type (yearling vs sub-yearling through an analysis of covariate least-square means; results of statistical contrasts are contained in S2 Table. Confidence intervals and comparison arrows for LS means (see Methods) are plotted for each life-history type in each season. Next, to explore changes in infectious agent abundance through time, we plot seasonal contrasts of the quantile-quantile distribution of infectious agent loads by life-history type (see Methods and Figure 2 for interpretations) and evaluated the slopes of quantile-quantile regressions of infectious agent loads between seasons for ocean-type and stream-type Chinook salmon. We regressed the 10, 25, 50, 75, 90th load percentiles within a season as a function of the same percentiles for the previous season using a bootstrap procedure to estimate the load at these percentiles and the confidence intervals associated with the quantile-quantile slope. The mean and 95% confidence limits (CL) of the quantile-quantile slopes were taken as the 50th percentile, and the 2.5th and 97.5th percentile, respectively. High loads are truncated when slope and CL (i.e. 97.5^th^ percentile) <1 and denoted by ** in the top left-hand corner of the plot.

| infectious agent | Trans-mission | Seasonal prevalence by life-history type | Seasonal contrast of quantile-quantile distribution of loads by life-history type |  |
| --- | --- | --- | --- | --- |
| c.b.cys | SW |  |   **    ** | |
|  |  |  |  | |
| ce.sha | FW |  |   **   | |
| env | SW |  |   **   | |
|  |  |  |  | |
| fa.mar | SW |  |    | |
| fl.psy | FW |  |    | |
| ic.hof | both |  |    | |
| ic.mul | FW |  |    | |
| ku.thy | SW |  |    | |
| lo.sal | both |  |   **    ** | |
| my.arc | FW |  |   **    **  ** | |
| pa.kab | SW |  |    | |
| pa.min | FW |  |   **    ** | |
| pa.pse | SW |  |   **   | |
| pa.the | SW |  |    | |
| prv | both |  |    | |
| re.sal | both |  |    | |
| rlo | both |  |    | |
| sch | SW |  |   **   | |
| sp.des | both |  |    | |
| te.bry | both |  |    | |
| vhsv | both |  |    | |
